# Age-Period-Cohort Analysis of Suicide Mortality in Japan: A 40-Year Nationwide and Prefectural-Level Study

**DOI:** 10.31662/jmaj.2025-0527

**Published:** 2026-02-27

**Authors:** Syeda Sabrina Easmin Shaba, Odgerel Chimed-Ochir, Yui Yumiya, Ami Fukunaga, Toru Yoshikawa, Badamtsetseg Jargalsaikhan, Tatsuhiko Kubo

**Affiliations:** 1 Department of Public Health and Health Policy, Graduate School of Biomedical and Health Sciences, Hiroshima University, Minamiku, Hiroshima, Japan; 2 Research Center for Overwork-Related Disorders, National Institute of Occupational Safety and Health, JAPAN (JNIOSH), Tama-ku, Kawasaki, Japan

**Keywords:** age-period-cohort analysis, suicide, Japan, global health, prefecture-level analysis

## Abstract

**Introduction:**

Suicide remains a major public health concern in Japan. Age-period-cohort analysis can differentiate the effects of age, date, and birthyear on an outcome. This study used an age-period-cohort analysis to analyze suicide mortality in all of Japan and in the 47 prefectures.

**Methods:**

Suicide mortality data from 1980 to 2021 were from the Global Burden of Disease database. Trends in the crude and age-standardized mortality rate were analyzed using Joinpoint regression. Age-period-cohort analysis was conducted using a Poisson model.

**Results:**

The suicide rate was consistently greater in men than in women. Analysis of different age groups indicated the highest rate was in men aged >80 years, although it decreased in this group over time. The suicide rate increased in the group aged 15 to 19 years, particularly in cohorts born after 1972. A strong period effect occurred from the late 1990s to early 2000s, particularly among working-age men in their 50s and 60s. There were some disparities among prefectures, in that Akita had the highest rate and Tokyo had the lowest.

**Conclusions:**

We identified key age-period-cohort effects of suicide in Japan. Economic downturns and generational differences affected suicide risk, and adolescents and middle-aged men had high risk.

## Introduction

In 2019, globally, suicide was the fourth leading cause of death among individuals aged 15-29 years ^(1)^. Although the suicide rate in Japan has recently declined, it is still higher than the global average of 9.0 per 100,000 per year ^(1)^. In 2019, Japan had the second-highest suicide rate (15.3 per 100,00) among all G7 nations ^(2)^. Preventive measures and increased societal awareness have led to gradual declines of the suicide rate in Japan over the past 2 decades ^(3)^, but suicide remains a leading cause of death for individuals aged 15-39 years ^(4)^.

Age-period-cohort (APC) analysis simultaneously examines the effects of age, period (calendar time), and cohort (birth year or generational effects) on an outcome ^(5)^, and is a valuable tool for identifying the effects of these factors on suicide ^(5)^. The results from APC analysis can help develop targeted prevention strategies by identifying high-risk age groups, the impact of historical events, and persistent vulnerabilities in certain cohorts.

Several previous studies have applied APC analysis to suicide mortality in Japan. However, these studies were largely confined to earlier time periods, typically ending around 2006-2015, and were primarily conducted at the national level or selected prefectures ^(6), (7), (8), (9)^. Consequently, recent societal changes including economic stabilization, demographic aging, and the coronavirus disease 2019 pandemic may have altered APC patterns of suicide across different regions of Japan. Moreover, comprehensive prefecture-level APC analyses covering the entire country are lacking.

To address these gaps, the present study aimed to analyze the long-term trends and generational patterns of suicide mortality in Japan overall and each of its 47 prefectures by conducting an APC analysis for the period of 1980-2021. By incorporating recent data and systematically assessing regional heterogeneity, this study seeks to provide updated and geographically granular evidence to inform more targeted suicide prevention strategies in Japan.

## Materials and Methods

### Data source

Data on suicide deaths in Japan from 1980 to 2021 were obtained from the 2021 Global Burden of Disease (GBD) study ^(10)^. For Japan, the GBD compiles suicide mortality data from the national vital registration system and uses the International Classification of Diseases (ICD) definition of suicide mortality as death caused by purposely self-inflicted poisoning or injury (ICD-10 codes: X60-X64.9, X66-X84.9, Y87.0; ICD-9 codes: E950-E959). Within the GBD analytical framework, data on suicide death applied redistribution of ill-defined or implausible causes of death (“garbage codes”) to valid causes based on established algorithms ^(11)^. Because Japan has a comprehensive and high-quality vital registration system, GBD-estimated suicide mortality rates closely mirror observed vital statistics ^(11)^.

Population data were also from the GBD 2021 database and the Statistics Bureau of Japan ^(12)^. Population and mortality data were structured into 16 5-year age groups (15-19, ..., 90-94 years) and by gender. Individuals younger than 15 years were excluded due to the rarity of suicide in this age group.

### Data analysis

First, the number of suicide deaths, crude mortality rate (CMR) from suicide, and age-standardized mortality rate (ASMR) from suicide were recorded for the years 1980, 1990, 2000, and 2021. The average annual changes in ASMR by gender were then shown for all of Japan and for each prefecture.

Second, Joinpoint regression software was used to assess trends in CMR and ASMR from 1980 to 2021. This method allowed identification of years with significant changes in the suicide rate and calculation of annual percentage change between those years ^(13)^.

Third, the mortality and population data were arranged into eight consecutive 5-year periods from 1982-1986 to 2017-2021 and 15 5-year age groups (described above). This structured data allowed visualization of the effects of age, period, and cohort on suicide mortality.

Fourth, APC analysis was performed using the Age-Period-Cohort Analysis Web Tool ^(14)^ of men and women in all of Japan and in each prefecture. This model assumes that the number of deaths followed a Poisson distribution, and models the expected number using a log-link function:

log(*E*[*Y*])=*Xβ* (1)

where X is the design matrix containing age, period, and cohort effects, and *β* represents the estimated regression coefficients. This transformation ensures that predicted values were positive, and is essential for analysis of count data. The model also incorporates an offset term to account for the size of the population at risk:

log(*E*[*Y*])=*Xβ*+log(offset) (2)

Because the APC model has an identification problem due to linear dependence, this method constructs a design matrix function that applies centering techniques and contrasts to mitigate collinearity. Thus, the model allowed evaluation of whether age, period, or cohort deviations significantly differed from zero based on the Wald test and confidence intervals. To allow for potential extra-Poisson variation, the age-period-cohort model applies implementation applies a deviance-based scale adjustment to the variance-covariance matrix, inflating standard errors when the residual deviance exceeds its degrees of freedom, while retaining Poisson point estimates ^(14)^.

The analysis further decomposed the results into age-specific deviations (suicide rate by age group), period deviations (period rate ratios [RRs] relative to a reference year), and cohort deviations (cohort RRs relative to a reference cohort). Rosenberg et al. ^(14)^ (2014) provided details of these methods.

Statistical analyses were performed using Joinpoint trend analysis software Version 5.3.0 (National Cancer Institute, National Institutes of Health, Maryland, USA), R version 4.3.2 (R Core Team, 2023) with custom scripts for age-period-cohort analysis developed by the US National Cancer Institute ^(15)^, and Microsoft Excel (2021).

## Results

### Descriptive analysis of suicide deaths for all of Japan and the 47 prefectures

Our descriptive analysis of suicide deaths in Japan from 1980 to 2021 revealed distinct trends among the 47 prefectures and all of Japan for men and women. For men, the total number of suicide deaths was 15,678 in 2021 (Table 1). The CMR rose from 23.89 per 100,000 in 1980 to a peak of 36.46 in 2000, and then declined to 25.20 in 2021. The ASMR had a similar trend, and was 18.86 in 2021 and then had an average annual change of −4.08% (95% confidence interval [CI], −6.21 to −1.94) from 1990 to 2021. Akita had the highest CMR and ASMR, and Tokyo had the lowest. Most regions had significant decreases in ASMR from 1990 to 2021, and the largest declines were in Iwate (−22% [95% CI, −29.07 to −14.24]) and Okinawa (−21.71% [95% CI, −29.47 to −14.64]). The largest increases were in Ibaraki (23.91% [95% CI, 14.81-33.16]) and Shizuoka (17.33% [95% CI, 8.61-27.73]).

**Table 1.** Descriptive Analysis of the Burden of Suicide Among Men between 1980 and 2021 in Japan.

	Number of Death				% of suicide death among all-cause death				CMR per 100,000				ASMR per 100,000				AAPC of ASMR, 1990-2021	
	1980	1990	2000	2021	1980	1990	2000	2021	1980	1990	2000	2021	1980	1990	2000	2021	%	95% CI
Japan	14,065	14,160	23,039	15,678	3.64	3.21	4.37	2.12	23.89	30.62	36.46	25.20	23.32	25.48	27.67	18.86	**−4.08**	**−6.21 to −1.94**
Akita	206	219	325	167	4.21	4.06	5.09	2.21	33.38	36.56	56.43	36.62	30.43	29.31	40.36	26.24	**−10.5**	**−18.96 to −1.12**
Aomori	231	223	347	193	4.16	3.61	4.72	2.04	30.59	27.21	48.24	32.82	29.86	22.61	36.35	24.60	−7.74	−17.13 to 0.80
Miyazaki	192	178	268	167	4.22	3.73	5.02	2.33	34.07	26.02	47.58	32.72	32.56	22.28	36.34	23.74	**−12.6**	**−21.28 to −1.35**
Saga	110	104	171	113	3.09	2.76	4.09	2.22	26.10	20.19	40.43	28.94	24.38	18.03	31.59	23.36	**12.14**	**0.31-25.35**
Shimane	142	132	179	110	3.81	3.45	4.22	2.19	36.65	23.21	48.20	33.34	32.63	20.20	35.92	23.10	**−15.4**	**−23.43 to −6.14**
Yamagata	170	166	243	155	3.32	3.06	3.82	2.03	27.41	24.79	39.56	29.72	25.23	20.94	29.46	22.97	5.92	−4.84 to 17.22
Nagasaki	213	196	295	193	3.36	2.99	4.12	2.12	27.41	29.64	40.49	30.98	26.62	24.52	31.68	22.91	2.81	−7.15 to 13.17
Kagoshima	282	264	362	232	3.56	3.26	4.02	2.14	32.77	21.58	42.23	30.65	30.63	18.44	32.13	22.74	**−10.8**	**−18.86 to −3.00**
Hokkaidō	721	685	1,134	724	3.98	3.32	4.60	2.10	25.76	20.17	41.07	29.04	25.20	17.83	31.29	22.73	7.2	−0.46 to 14.95
Niigata	338	364	567	327	3.66	3.68	4.77	2.14	27.62	28.84	46.25	30.39	26.13	27.80	34.14	22.34	**−8.88**	**−16.61 to −0.56**
Fukushima	238	236	394	267	3.02	2.78	3.84	2.08	23.43	23.27	37.12	29.31	22.25	20.03	29.07	22.19	**13.57**	**4.87-23.66**
Ibaraki	287	293	533	400	3.21	2.89	4.23	2.24	22.06	33.39	35.13	27.59	21.47	27.26	27.20	22.10	**23.91**	**14.81-33.16**
Okinawa	160	176	282	214	5.35	5.20	6.48	3.02	28.67	18.00	42.86	28.92	30.44	16.89	36.82	21.76	**−21.7**	**−29.47 to −14.64**
Yamanashi	100	106	165	115	3.08	3.02	4.09	2.15	24.95	24.29	37.01	28.71	23.46	21.46	28.15	21.65	3.39	−6.21 to 14.47
Gunma	228	231	363	273	3.53	3.22	4.12	2.19	24.46	24.61	35.65	28.09	23.34	21.20	27.27	21.29	6.31	−1.74 to 16.22
Iwate	237	233	339	184	4.45	3.92	4.92	2.10	33.51	23.33	48.76	31.29	31.65	19.51	36.81	21.26	**−22**	**−29.07 to −14.24**
Wakayama	149	154	219	129	3.27	3.16	4.06	1.95	27.80	27.46	42.33	29.42	25.18	22.03	31.48	21.09	**−11.7**	**−20.48 to −2.07**
Kōchi	143	125	170	96	3.54	3.15	3.91	1.88	35.15	18.54	43.34	28.99	31.23	16.66	31.84	21.07	**−17**	**−25.54 to −6.85**
Yamaguchi	231	209	312	187	3.44	2.95	3.92	1.98	29.78	25.65	42.30	29.12	27.79	21.06	31.36	20.98	−4.78	−12.73 to 4.34
Tottori	83	86	115	74	3.23	3.07	3.70	1.98	27.97	29.39	38.44	27.52	25.47	23.87	29.45	20.93	**−10.7**	**−20.89 to −0.94**
Miyagi	230	256	432	299	3.48	3.33	4.55	2.29	21.91	22.90	36.56	26.28	21.20	18.63	28.83	20.48	2.45	−6.46 to 12.01
Ehime	207	200	279	178	3.35	3.03	3.73	1.93	28.11	26.66	38.76	27.70	26.40	23.01	29.29	20.42	**−9.66**	**−19.03 to −1.17**
Tokushima	114	95	132	93	3.11	2.54	3.20	1.77	28.21	20.61	32.98	26.93	25.76	17.62	24.66	20.18	4.88	−5.80 to 16.41
Hyōgo	650	610	995	676	3.70	3.05	4.36	2.15	25.33	22.05	36.54	25.67	25.11	18.92	27.69	20.11	2.21	−4.86 to 8.83
Toyama	143	144	220	140	3.47	3.12	4.15	2.01	26.28	26.76	39.89	27.49	24.51	21.69	28.85	20.04	−5.42	−14.40 to 6.41
Ōita	161	153	226	139	3.13	2.87	3.83	1.91	26.93	30.82	38.56	25.77	24.83	26.67	28.86	19.79	−6.06	−14.42 to 4.09
Kumamoto	224	224	326	220	3.10	2.96	3.91	1.99	25.73	20.83	36.33	26.32	24.22	17.46	27.91	19.69	−7.05	−14.63 to 1.39
Shizuoka	382	359	605	464	3.47	2.86	3.91	2.08	21.99	23.53	31.93	25.59	21.73	19.25	23.95	19.61	**17.33**	**8.61-27.73**
Fukuoka	589	626	981	634	3.68	3.45	4.74	2.29	26.21	21.45	40.38	25.67	25.65	18.12	31.03	19.42	**−15.6**	**−21.96 to −8.75**
Tochigi	216	228	363	254	3.38	3.17	4.27	2.16	23.79	26.20	35.76	26.02	23.19	21.19	27.54	19.33	−4.29	−12.12 to 3.61
Hiroshima	347	345	501	341	3.51	3.14	3.92	2.16	25.38	22.90	35.33	24.79	24.62	19.67	26.61	19.00	**−8.98**	**−16.14 to −1.50**
Nagano	236	246	392	257	2.90	2.81	3.81	1.98	22.86	19.99	35.54	25.30	21.21	17.45	27.03	18.84	1.11	−8.15 to 10.86
Okayama	217	211	306	218	2.91	2.60	3.41	1.89	23.45	24.55	32.09	23.68	21.70	20.83	24.19	18.71	1.78	−9.27 to 12.68
Ōsaka	1,060	1,063	1,809	1,078	4.30	3.63	5.23	2.08	24.71	17.91	41.37	25.09	25.49	16.21	30.70	18.70	**−12.9**	**−18.14 to −7.13**
Gifu	215	220	353	247	3.15	2.95	3.89	1.99	21.98	24.40	33.80	25.36	22.06	20.88	25.76	18.61	2.68	−6.58 to 12.64
Chiba	470	511	992	774	3.80	3.26	4.72	2.20	19.32	22.87	32.75	24.41	20.16	18.81	24.86	18.37	**13.36**	**4.83-22.33**
Kagawa	114	117	171	115	2.99	2.67	3.51	1.89	23.25	25.17	34.15	24.69	21.43	21.18	26.18	18.20	−6.74	−16.89 to 4.65
Shiga	109	124	203	154	2.87	2.95	4.10	2.27	20.22	19.41	30.08	21.82	19.77	16.71	23.80	17.52	−2.78	−14.18 to 7.99
Nara	129	135	213	149	3.22	2.87	3.88	1.83	21.57	22.21	30.29	23.60	21.20	18.38	23.21	17.32	−0.73	−9.94 to 10.74
Mie	188	185	296	216	2.95	2.58	3.62	1.92	22.42	31.58	32.16	24.70	21.33	27.17	24.69	17.26	−1.09	−10.48 to 9.33
Saitama	541	595	1,080	856	4.14	3.50	4.77	2.09	19.32	34.42	30.34	23.07	21.26	27.29	23.37	17.00	0.64	−6.40 to 7.47
Kanagawa	688	772	1,382	1,043	4.14	3.52	4.73	2.19	19.09	31.39	31.58	22.37	20.34	25.38	24.21	16.79	0.8	−6.19 to 7.99
Kyōto	291	278	485	270	3.40	2.90	4.32	1.84	22.99	22.70	37.43	21.69	22.09	19.99	27.75	16.77	−9.02	−17.27 to 0.52
Ishikawa	123	127	202	127	3.26	2.90	3.93	1.89	22.14	22.89	34.74	22.82	21.18	19.66	26.38	16.73	**−11.6**	**−19.76 to −1.86**
Fukui	88	94	141	88	3.13	2.86	3.73	1.78	22.44	22.53	34.25	23.21	20.83	19.54	25.34	16.43	**−12.7**	**−21.74 to −1.18**
Aichi	610	621	1,093	826	3.58	3.07	4.30	2.12	19.18	18.15	30.59	21.64	20.00	16.46	23.90	16.37	−0.55	−8.41 to 6.52
Tōkyō	1,262	1,240	2,079	1,503	3.95	3.20	4.45	2.26	21.19	28.44	34.13	21.39	20.73	23.44	25.42	16.10	**−8.64**	**−14.33 to −2.38**

Prefectures are ranked based on their ASMR for the year 2021. Bold numbers are statistically significant. AAPC: average annual percentage change; ASMR: age-standardized mortality rate; CMR: crude mortality rate.

For women, the total number of suicide deaths was 6,626 in 2021 (Table 2). The ASMR was 7.56 in 2021, and the average annual change from 1980 to 2021 was −27.74% (95% CI, −29.50 to −25.96). Akita had the highest mortality rate, and Okayama had the lowest. All prefectures had significant decreases in average annual ASMR from 1990 to 2021, and the largest decreases were in Hiroshima (−45.58% [95% CI, −50.34 to −40.63]) and Niigata (−42.22% [95% CI, −47.95 to −35.67]).

**Table 2.** Descriptive Analysis of the Burden of Suicide Among Women between 1980 and 2021 in Japan.

	Number of Death				% of suicide death among all-cause death				CMR per 100,000				ASMR per 100,000				AAPC of ASMR, 1990-2021	
	1980	1990	2000	2021	1980	1990	2000	2021	1980	1990	2000	2021	1980	1990	2000	2021	%	95% CI
Japan	8,693	8,727	9,279	6,626	2.65	2.35	2.12	0.95	14.34	15.00	14.10	10.12	13.04	10.81	9.97	7.56	**−27.74**	**−29.50 to −25.96**
Akita	130	142	142	76	3.20	3.12	2.61	0.85	19.55	21.69	22.25	15.00	16.56	14.69	13.85	9.41	**−35.99**	**−42.77 to −29.14**
Iwate	151	159	146	83	3.42	3.18	2.54	0.91	20.21	13.64	19.53	13.23	17.63	9.83	12.56	9.12	**−39.54**	**−46.52 to −32.69**
Tochigi	153	161	166	110	2.76	2.62	2.35	1.00	16.57	17.15	16.15	11.33	14.88	12.31	11.44	8.82	**−30.16**	**−36.48 to −23.14**
Fukushima	157	170	162	104	2.27	2.36	1.90	0.83	14.67	16.42	14.62	11.16	12.76	12.32	10.10	8.71	**−23.16**	**−30.49 to −14.61**
Ōsaka	618	583	684	533	3.09	2.43	2.47	1.19	14.21	11.40	14.98	11.44	14.22	9.57	10.92	8.69	**−17.55**	**−23.14 to −11.74**
Hokkaidō	394	376	424	317	2.83	2.38	2.25	0.94	13.61	13.38	14.10	11.40	13.05	10.54	9.82	8.64	**−12.63**	**−19.46 to −5.52**
Yamagata	122	115	111	66	2.68	2.32	1.92	0.79	18.50	14.37	16.92	11.94	14.91	10.62	10.77	8.54	**−26.82**	**−36.20 to −18.61**
Niigata	282	282	269	141	3.49	3.24	2.69	0.91	21.95	10.15	20.77	12.41	18.41	9.06	12.66	8.49	**−42.22**	**−47.95 to −35.67**
Aomori	119	125	128	78	2.68	2.47	2.12	0.83	14.73	14.39	16.18	11.87	13.49	10.57	11.12	8.45	**−28.85**	**−36.59 to −20.34**
Tōkyō	742	730	900	744	2.84	2.33	2.39	1.23	12.70	14.68	14.77	10.23	11.88	10.34	10.69	8.31	**−14.24**	**−19.39 to −8.52**
Ehime	116	117	115	70	2.23	2.06	1.75	0.75	14.43	12.63	14.36	9.93	12.15	9.70	9.85	8.02	**−24.12**	**−31.57 to −15.99**
Saitama	330	391	455	383	3.10	2.86	2.57	1.14	12.07	18.06	13.04	10.22	12.91	12.13	9.79	7.97	**−25.13**	**−30.84 to −18.74**
Miyagi	143	156	164	125	2.46	2.48	2.15	0.98	13.25	16.35	13.39	10.47	12.26	11.47	9.50	7.97	**−24.3**	**−31.77 to −16.04**
Nara	91	90	94	72	2.57	2.14	1.92	0.94	14.34	12.65	12.30	10.23	13.09	9.13	8.81	7.86	**−19.88**	**−27.63 to −11.20**
Nagano	170	185	177	107	2.35	2.36	1.96	0.81	15.51	12.38	15.33	10.10	12.87	9.81	10.32	7.83	**−31.74**	**−37.59 to −25.42**
Kōchi	76	67	63	40	2.18	1.87	1.62	0.74	17.17	11.30	14.51	10.87	13.53	9.51	9.54	7.82	**−24.1**	**−33.02 to −14.12**
Gunma	171	166	167	111	2.98	2.68	2.28	0.93	17.81	12.64	16.06	11.30	15.85	9.89	10.90	7.76	**−36.96**	**−42.72 to −30.52**
Miyazaki	100	97	101	63	2.67	2.43	2.17	0.83	16.30	12.43	16.02	11.17	14.33	9.31	10.59	7.69	**−34.1**	**−40.96 to −26.18**
Shimane	82	75	66	39	2.58	2.33	1.91	0.82	19.62	16.22	16.28	11.17	15.36	12.62	11.40	7.64	**−37.01**	**−44.00 to −29.32**
Hyōgo	434	391	407	295	2.92	2.30	2.11	0.98	16.19	12.54	13.95	10.18	14.75	9.18	10.01	7.56	**−29.03**	**−35.23 to −23.32**
Chiba	278	319	372	313	2.69	2.53	2.24	1.06	11.62	13.71	12.44	9.76	11.89	9.98	9.22	7.55	**−21.15**	**−26.53 to −14.78**
Mie	124	127	133	93	2.20	2.00	1.88	0.88	13.93	15.48	13.66	10.23	11.88	11.67	9.39	7.54	**−23.09**	**−31.48 to −14.17**
Kanagawa	352	445	539	471	2.68	2.61	2.48	1.13	10.20	15.01	12.73	9.98	10.49	10.31	9.47	7.43	**−21.9**	**−27.24 to −16.83**
Toyama	101	102	98	56	2.80	2.56	2.13	0.83	17.37	17.36	16.60	10.44	14.69	11.68	10.83	7.40	**−39.87**	**−46.68 to −32.59**
Ibaraki	176	194	208	142	2.33	2.29	2.02	0.87	13.41	21.12	13.64	9.81	12.32	15.09	9.93	7.40	**−29.78**	**−35.78 to −23.07**
Wakayama	107	101	94	58	2.65	2.32	1.96	0.90	18.57	14.60	16.38	11.82	15.31	10.28	10.92	7.39	**−39.22**	**−45.94 to −31.89**
Fukuoka	328	322	347	258	2.46	2.12	1.93	0.92	13.67	16.06	13.01	9.42	12.16	11.94	9.37	7.09	**−26.84**	**−32.85 to −20.86**
Kagawa	83	74	72	50	2.41	1.96	1.60	0.80	15.61	12.69	13.33	10.16	12.71	9.14	8.90	7.08	**−27.99**	**−35.55 to −19.87**
Yamaguchi	145	123	126	69	2.61	2.04	1.76	0.69	17.19	13.79	15.33	9.70	14.29	9.85	9.89	7.03	**−31.56**	**−38.82 to −23.97**
Gifu	171	174	170	101	2.90	2.69	2.23	0.85	16.66	14.30	15.42	9.81	15.33	10.66	10.52	6.96	**−41.69**	**−46.50 to −36.24**
Ōita	99	92	92	52	2.19	1.93	1.72	0.73	14.96	15.68	14.09	8.82	12.64	11.88	9.44	6.87	**−30.26**	**−37.73 to −22.50**
Nagasaki	113	105	102	67	2.06	1.83	1.61	0.71	13.34	21.75	12.43	9.59	11.54	14.70	8.88	6.77	**−27.24**	**−34.76 to −18.68**
Yamanashi	65	64	62	39	2.35	2.14	1.76	0.81	15.52	12.97	13.58	9.43	13.43	10.53	9.59	6.74	**−36.54**	**−43.19 to −29.23**
Okinawa	66	64	77	65	2.37	2.17	2.13	1.05	11.56	12.16	11.38	8.63	11.42	10.65	9.43	6.65	**−26.58**	**−34.49 to −18.35**
Kagoshima	166	146	139	79	2.23	1.93	1.68	0.70	17.16	14.67	14.40	9.33	14.20	10.86	9.56	6.64	**−38.62**	**−44.71 to −31.42**
Aichi	428	424	468	339	2.94	2.51	2.27	0.97	13.50	12.51	13.12	8.85	13.61	10.26	9.56	6.59	**−35.74**	**−40.02 to −31.04**
Kyōto	192	199	192	125	2.52	2.26	1.97	0.90	14.59	13.44	13.85	9.19	12.85	10.52	9.80	6.55	**−39.67**	**−44.98 to −33.70**
Saga	60	58	56	38	1.90	1.71	1.49	0.74	13.00	12.94	11.85	8.89	10.96	10.16	8.18	6.45	**−28.96**	**−37.51 to −19.87**
Tottori	49	48	44	27	2.22	1.98	1.61	0.67	15.41	17.50	13.38	9.30	12.64	12.16	9.24	6.37	**−38.4**	**−45.81 to −30.59**
Tokushima	73	64	57	40	2.37	1.95	1.56	0.69	16.72	12.29	13.01	10.69	13.70	9.69	8.42	6.36	**−38.67**	**−46.26 to −30.55**
Shizuoka	217	219	218	160	2.35	2.06	1.70	0.76	12.15	14.45	11.23	8.63	11.18	10.36	7.96	6.19	**−30.46**	**−36.78 to −23.93**
Kumamoto	136	126	134	81	2.12	1.83	1.74	0.72	14.14	13.45	13.39	8.73	11.86	9.80	9.39	6.16	**−32.59**	**−39.31 to −25.63**
Fukui	60	59	55	35	2.23	1.97	1.68	0.70	14.41	15.48	12.70	8.91	11.98	11.33	8.56	6.07	**−39.17**	**−46.69 to −31.46**
Shiga	82	82	81	60	2.41	2.15	1.92	0.92	14.62	11.56	11.78	8.34	12.93	8.90	8.48	5.90	**−41.95**	**−47.92 to −35.81**
Hiroshima	230	213	199	125	2.75	2.29	1.89	0.78	16.11	13.83	13.20	8.58	14.16	10.65	9.15	5.80	**−45.58**	**−50.34 to −40.63**
Ishikawa	78	77	76	50	2.12	1.95	1.63	0.73	13.19	13.64	12.36	8.45	11.59	10.46	8.41	5.62	**−38.77**	**−44.41 to −31.70**
Okayama	133	129	126	74	2.11	1.86	1.62	0.67	13.54	12.30	12.22	7.46	11.30	9.08	8.64	5.36	**−41.37**	**−47.29 to −34.87**

Prefectures are ranked based on their ASMR for the year 2021. Bold numbers are statistically significant. AAPC: average annual percentage change; ASMR: age-standardized mortality rate; CMR, crude mortality rate.

### Trends of CMR and ASMR for all of Japan and the 47 prefectures

**Figure 1.**
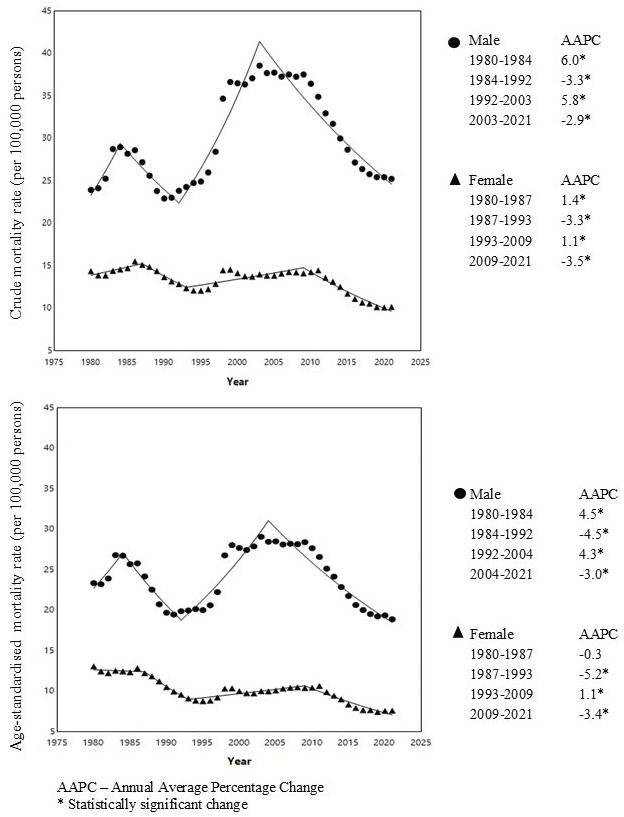
Trend of crude mortality rates (A) and age-standardized suicide rates (B) of suicide with their changes during 1982 and 2021 in Japan.

Our nationwide analysis of the trends in CMR (Figure 1A) and ASMR (Figure 1B) in men and women from 1980 to 2021 demonstrated four distinct phases, each with three significant points of change. For men, there was a significant increase in the average annual percentage change from 1980 to 1984 (CMR: 6.0%, ASMR: 4.5%) and from 1992 to 2003 (CMR: 5.8%, ASMR: 4.3%). For women, the CMR increased 1980 to 1987 and from 1993 to 2009, and the ASMR increased from 1993 to 2009, but these changes were much smaller. Our prefecture-level analysis indicated a similar timing of increases in the suicide rate of men and women in all prefectures except Shimane (Supplementary Figure 1). For men, this increase occurred between 1996 and 1998, and for women this increase occurred in most prefectures between 1995 and 2003.

### Visualization of the effects of APC on suicide mortality

**Figure 2.**
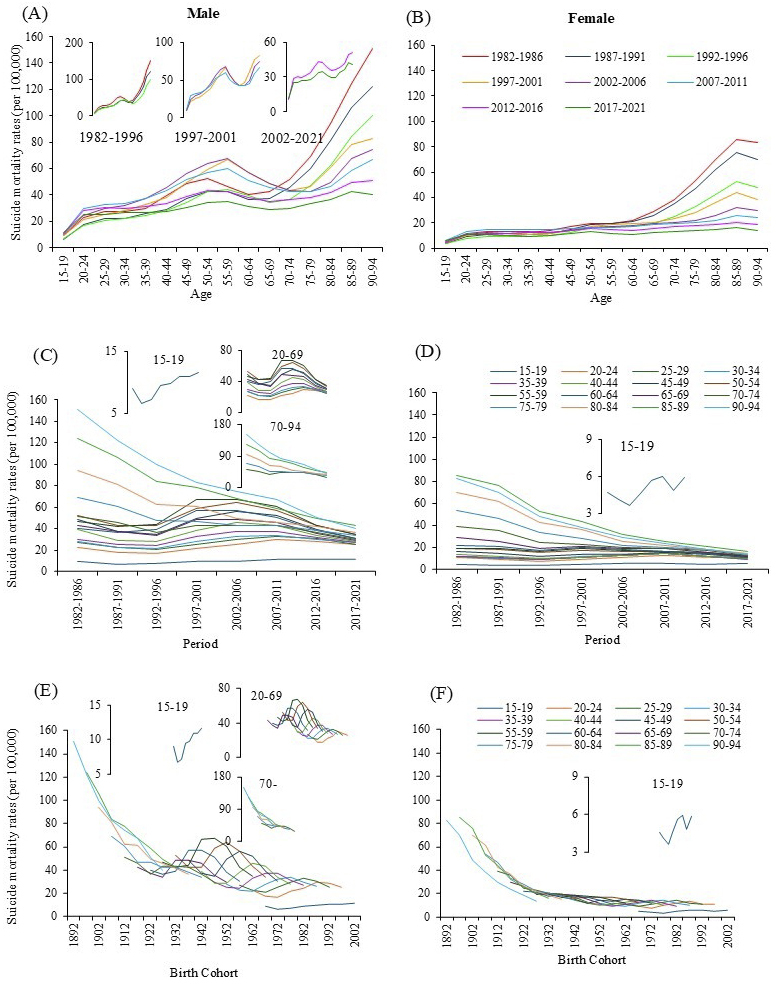
Trends of age-specific (A, B), period-specific (C, D) and cohort-specific (E, F) suicide death rates in Japan during 1982-2021 in men and women.

We then analyzed changes in the age-specific, period-specific, and cohort-specific suicide mortality rate of men (Figure 2A, C, and E) and women (Figure 2B, D, and F). For both genders, the periods with the lowest suicide mortality were for individuals aged 15-40 years during 1992-1996, and for individuals aged >40 years during 2017-2021. In addition, individual older than 80 had the highest rates during the entire study period.

Age-specific analysis of men (Figure 2A) indicated changes during three distinct time periods: (*i*) a sharp increase in those >70 years from 1982 to 1996; (*ii*) a peak for the 55-59 group, and a decrease for those aged 60-75 years from 1997 to 2011; and (*iii*) slight decreases for most age groups from 2012 to 2021. Period-specific analysis of men (Figure 2C) also indicated three distinct periods: (*i*) the group aged 15-19 years had an increasing rate throughout the study period; (*ii*) those aged 20-69 years had a stable rate until 1996, a sharp increase during 1997 to 2001, and then a decrease; and (*iii*) those >70 years had a consistent decrease over time. Cohort-specific analysis of men (Figure 2E) indicated that those born before 1930 had significantly higher rates in all age groups. The group aged 15-19 years had a concerning pattern, in that the suicide rate sharply increased for individuals born after 1972. In contrast, the suicide rates of those >70 years had a consistent decline in all birth cohorts.

The age-specific analysis of women (Figure 2B) had a more consistent pattern, in that the suicide mortality rate increased steadily with age during all time periods. The period-specific analysis of women (Figure 2D) showed that suicide remained low until the age of 70, and the highest rate was in the group aged 85-89 years. In addition, those who were aged 20-36 years and the 15-19 years age group had slight increases in suicide mortality during 1997-2001. The trends in cohort-specific analysis of women (Figure 2F) were similar to those of men, in that cohorts born before 1930 had significantly higher rate of suicide mortality. The suicide mortality rate had a steady decline across successive birth cohorts, and the decline was greatest in the older cohorts (1892-1922), which started at a higher baseline. However, for the 15-19 age group, the suicide mortality rate increased sharply for those born between 1977 and 1992. Although there was a decline in the 1992-1996 cohort, this trend changed, and the rate was higher in the cohort born in 1997. The mortality rates for women aged 15-44 years were highest during 2007 to 2011, and women >50 years had the highest rate from 1982 to 1986.

### APC analysis for all of Japan

**Figure 3.**
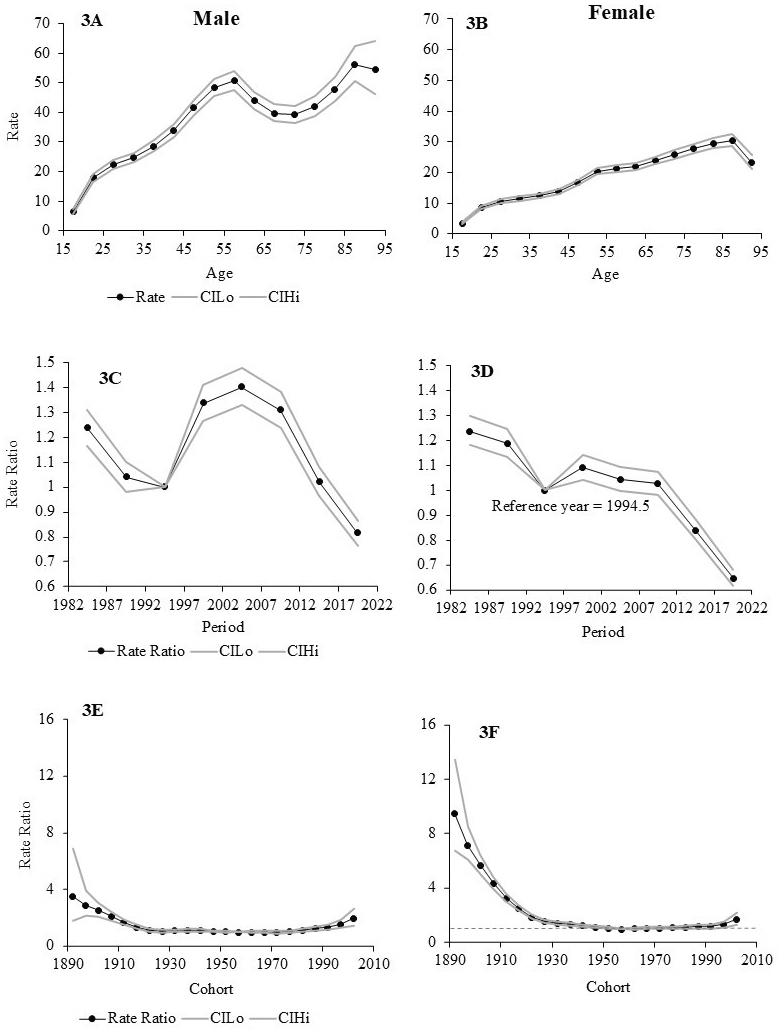
Age (A, B), period (C, D), and cohort effects (E, F) on the suicide death in Japan during 1982-2021.

We then performed an APC analysis of suicide deaths in Japan with adjustment for age, period, and cohort effects, and presented RRs to quantify the effect relative to reference groups for men (Figure 3A, C, and E) and women (Figure 3B, D, and F). For both genders, we selected 37.5 years as the reference age, 1994.5 as the reference period, and 1957 as the reference cohort, based on when the suicide rate began to increase significantly.

Analysis of the effect of age in men indicated the rate generally increased with age, although there were some fluctuations (Figure 3A). The lowest rate was in the 17.5 years age group (6.48 per 100,000 [95% CI, 5.81-7.22]), there was then a peak for the 57.5 years age group (50.65 per 100,000 [95% CI, 47.64-53.84]), a slight decline and then an increase for the 72.5 years age group. Period analysis (Figure 3C) indicated the RR increased significantly from 1994.5 to a maximum in 2004.5 (RR: 1.40 [95% CI, 1.33-1.48]). This was followed by a gradual decline, and the lowest RR was in 2019.5 (RR: 0.81 [95% CI, 0.77-0.86]). Cohort analysis (Figure 3E) indicated the 1892 cohort had the highest RR (3.54, 95% CI 1.82-6.86) and that cohorts between 1922 and 1992 had no significant differences from the reference cohort. However, cohorts after 1992 had significantly higher RRs (1.35, 95% CI 1.18-1.55). More specifically, the 1997 cohort had an RR of 1.57 (95% CI, 1.32-1.87) and the 2002 cohort had an RR of 1.96 (95% CI, 1.45-2.64).

Analysis of the effect of age in women (Figure 3B) indicated the rate also increased with age, and there was a peak of 30.45 per 100,000 (95% CI, 28.55-32.47) at age 87.5 years. Period analysis (Figure 3D) indicated the highest RR was in 1984.5 (RR: 1.24 [95% CI, 1.18-1.30]), and this was followed by a decrease. Cohort analysis (Figure 3F) indicated a maximum for the 1892 cohort (RR: 9.52 [95% CI, 6.73-13.47]), and that the RR between 1930 and the 1990s approached 1.0. However, there was a slight upward trend for cohorts in 1992 (RR: 1.18 [95% CI, 1.04-1.33]), 1997 (RR: 1.31 [95% CI, 1.11-1.53]), and 2002 (RR: 1.70 [95% CI, 1.32-2.19]).

### APC analysis for the 47 prefectures

We also performed adjusted APC analysis for each of the 47 prefectures (Supplementary Figure 2). The trends were generally similar to those in the nationwide analysis, but there were some exceptions. For example, although the nationwide analysis showed a decrease in the rate for the group aged 85 to 89 years, the rate increased for this age group in Ehime, Ibaraki, Kagawa, Nagano, Nara, Oita, Okayama, Okinawa, Shiga, Tottori, and Yamaguchi. Based on the reference period of 1994.5, Niigata, Gifu, Mie, Gunma, and Shiga had more pronounced and sharper declines in the RR after 2000. Compared to the reference cohort of 1957, the national trend showed an increase in the RR for men and women in cohorts born after 1972; however, more recent female cohorts in Aomori, Fukui, Ishikawa, Wakayama, and Tokushima had lower RRs.

## Discussion

The current APC analysis leverages data from 1980 to 2021, extending a decade beyond earlier analyses, and uniquely covers all 47 prefectures. This expanded scope has revealed several new insights that contrast with or build upon the earlier findings. By incorporating prefecture-level detail, it also uncovers regional variations that national studies could not see.

Our results showed the suicide rate was typically two times higher in men than in women, in agreement with previous studies ^(16), (17)^.

The current APC analysis of the effect of age revealed a sharp decline in the suicide rate at around age 60 years, and an increase after age 75 years. The decrease might be because retirement initially increases happiness and decreases psychological stress ^(18)^. The suicide rates of individuals aged 20-69 years remained stable until 1996, after which they increased sharply, and then declined after 2006. This pattern is most likely linked to the economic conditions of Japan, particularly the financial crisis of the late 1990s and subsequent economic recovery ^(8), (19)^. Our findings also showed that individuals >80 years consistently had a higher suicide mortality rate than other age groups. This is likely due to the factors such as financial hardship, family problems, and health issues, including a higher prevalence of chronic illnesses (cancer and chronic obstructive pulmonary disease) in the elderly ^(20), (21), (22)^. However, our findings also indicated a decline in the suicide rate among the oldest age groups overtime for all birth cohorts. This might be because of improvements in healthcare, more comprehensive public health interventions, and stronger family and community networks ^(23), (24), (25)^. Male and female Japanese adolescents (aged 15-19 years) had increased suicide rates over time, suggesting this age group needs more attention. A key new finding is that the suicide rate for men in this age group was more pronounced for those born after 1972, during Japan’s “baby boom”. The intensified academic pressure experienced by this cohort may be responsible for its greater suicide rate. Thus, these individuals likely experienced academic pressure, bullying, stigmas about seeking mental healthcare, environmental difficulties and family conflicts, addition to gaming, and internet dependency ^(26), (27), (28)^. This underscores the importance of early interventions and the need for policy measures that address the rising suicide rates in this age group.

Our analysis of the effect of period (calendar time) identified three distinct periods: before the economic crisis (1982-1996), during the economic crisis (1997-2011), and after the economic crisis (2012-2021). During the economic crisis period, particularly the 1997-1998 financial crisis, suicide rates increased, especially among working-age people who experienced unemployment ^(29)^, bankruptcy, and decreased income ^(19)^. In particular, those aged 55-59 years were most likely to be middle-management workers who experienced intense pressure at work, overwhelming responsibilities, and this often culminated in *karojisatsu* (suicide from overwork) ^(9), (30)^. Japan subsidized and provided allowances to companies from 1996 to 2006 if a manager passed away, and this may have inadvertently led to a higher suicide rate. This financial incentive could have created unintended pressure, particularly during economic downturns, and struggling companies might have perceived the death of a manager as providing some financial relief ^(31)^. The suicide rate in Japan decreased after the economic crisis. This may be because the 2006 amendment to the Money Lending Business Act (which protected debtors and curbed heavy debts) helped to reduce the financial stress experienced by middle-aged and older men. Measures such as the 2006 Basic Act on Suicide Prevention, the 2007 Suicide Comprehensive Measures Charter, the Basic Act on Measures Against Alcohol-related Harm, and workplace reforms (2015 Stress Check Program and overtime limits in 2019) may have also contributed to this decline ^(32)^.

Another key finding is the identification of a recent cohort effect indicating increased suicide risk among those born after 1992, essentially the cohorts now in their teens and 20s. Earlier APC studies suspected rising risk in younger generations ^(9)^ but could not definitively assess cohorts born in the 1990s. In the new 1980-2021 analysis, these cohorts have now entered young adulthood (reaching their 20s and early 30s by the 2010s/early 2020s), and the data show a clear uptick in suicide rates for these groups. In other words, these cohorts in Japan are experiencing higher suicide mortality than prior cohorts did at the same age. This is a novel insight, whereas previous studies documented cohort increases up through those born in the 1970s-1980s.

Another major contribution of the current study is the demonstration of regional disparities in age-period-cohort trends that were not visible in national-level data. The suicide rate in Japan varies among prefectures, and the causes of suicide are complex and multifaceted, making it challenging to identify region-specific factors. A previous study has identified gender-specific factors contribute to these disparities, where men’s rates are affected by the number of psychiatric social workers, household income, and patient rates, while women’s rates are influenced by access to counseling services, birth rates, and overtime work hours ^(33), (34)^. Additionally, differences in climate and urbanization may influence these regional variations ^(35), (36), (37)^. There is some evidence that urbanization in Japan was associated with decreased male suicide mortality from 1970 to 1990 ^(37)^. However, Japan is considered a highly developed nation, and the similarity of economic and human development among prefectures presents a unique challenge in identifying the reasons for disparities in the suicide rate.

Our analysis showed a high suicide rate in Akita, in agreement with previous studies, and this was possibly due to its remoteness, low population density ^(17)^, lack of job opportunities, harsh winters, and high percentage of elderly ^(36)^. Additionally, the higher prevalence of depressive disorders and economic problems also contribute to the high rate of suicide in Akita ^(33), (35)^. In contrast, Okayama had the lowest rate of suicide by women, possibly because it has a mild climate, many advantages of urban living, and better access to healthcare ^(36)^. Tokyo also had a lower rate of suicide by men, and this may be linked to the higher social trust and capital in this prefecture ^(38)^.

Ibaraki and Shizuoka had the greatest increases in average annual ASMR. However, the reasons for the increasing rates in these prefectures remain unclear.

Our results also have certain policy implications. Japan’s suicide rate recently declined, but remains higher than many other G7 countries, underscoring the need for preventive measures. The high vulnerability of the group aged 15-19 years highlights the importance of targeting adolescents. Special attention is also needed for certain prefectures, such as Akita, in which the historically high suicide rate has decreased but remains high. The rising suicide rates in Ibaraki and Shizuoka require urgent investigation and targeted action.

The strengths of this study include the use of four decades of complete data from all 47 prefectures in Japan, allowing analysis of regional variations and patterns. However, a limitation is that APC analysis overlooks contextual, socioeconomic, and cultural factors that may change over time, and these could influence trends. Additionally, it was challenging to identify the specific factors behind prefecture-level changes.

### Conclusions

This nationwide APC analysis of suicide mortality in Japan (1980-2021) identified distinct temporal, generational, and regional patterns among men and women. Although overall suicide rates have declined in recent years, particularly among older adults, worrying increases were observed among adolescents aged 15-19, especially those born after 1972. Period effects closely aligned with major economic events, underscoring the sensitivity of suicide mortality to macroeconomic conditions. Cohort effects further revealed persistent vulnerability among younger generations. Substantial regional disparities remain, with prefectures such as Akita maintaining high rates and others, including Ibaraki and Shizuoka, showing rising trends. These findings highlight the need for age-specific, cohort-sensitive, and regionally tailored suicide prevention strategies, with particular emphasis on adolescents and economic downturn preparedness.

## Article Information

### Author Contributions

Study concept and design: Odgerel Chimed-Ochir; Data management: Syeda Sabrina Easmin Shaba, Odgerel Chimed-Ochir, Badamtsetseg Jargalsaikhan; Data analysis: Syeda Sabrina Easmin Shaba; Interpretation of data: Syeda Sabrina Easmin Shaba, Odgerel Chimed-Ochir; Manuscript development: Syeda Sabrina Easmin Shaba; Manuscript review: Odgerel Chimed-Ochir, Yui Yumiya, Ami Fukunaga, Toru Yoshikawa, Tatsuhiko Kubo. Study supervision: Odgerel Chimed-Ochir. All authors reviewed and approved the final version of the manuscript.

### Conflicts of Interest

None

### Ethical Approval and Consent to Participate

Publicly available data is used, thus, ethical approval and consent to participate is not applicable to this research.

## Supplement

Supplementary Material
