# Supplementary material for: Age-Period-Cohort Analysis of Suicide Mortality in Japan: A 40-Year Nationwide and Prefectural-Level Study
Source: JMA J. 2026 Feb 27;9(3):606–16. doi: 10.31662/jmaj.2025-0527 (PMC13246249; doi:10.31662/jmaj.2025-0527)
Supplement: Supplementary Material [file 2433-3298-9-3-0606-s001.pdf]

Supplementary Figure 1. Prefectural trend of suicide rates and their changes during 1980 and 2021 in Japan.

1. Aichi Prefecture

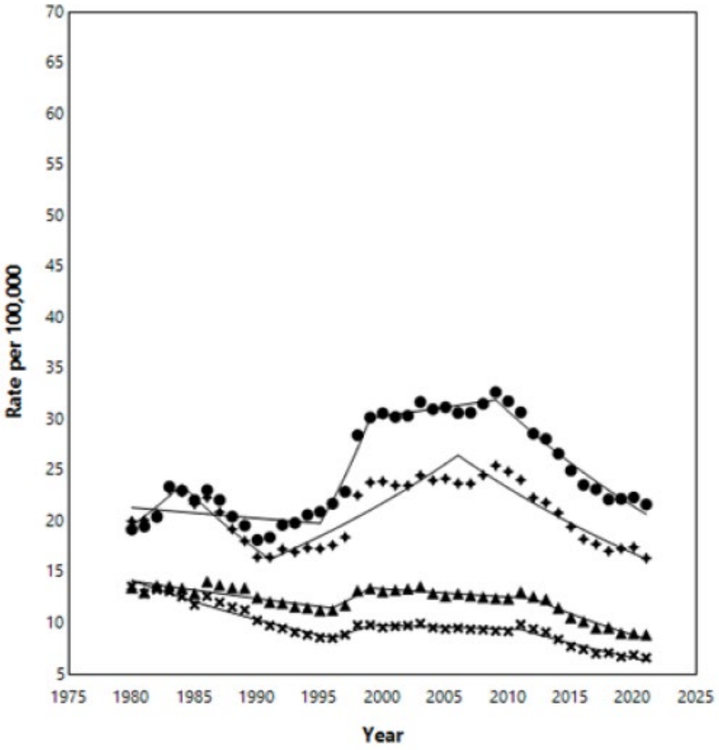

● **Male / All ages – 3 joinpoints**
1980–1995 APC = −0.5
1995–1999 APC = 11.1*
1999–2009 APC = 0.6
2009–2021 APC = −3.5*

▲ **Female / All ages – 3 joinpoints**
1980–1996 APC = −1.3*
1996–1999 APC = 5.4*
1999–2012 APC = −0.6
2012–2021 APC = −4.2*

✚ **Male / Age-standardized – 3 joinpoints**
1980–1984 APC = −4.8*
1984–1991 APC = −5.2*
1991–2006 APC = 3.3*
2006–2021 APC = −3.2*

✕ **Female / Age-standardized – 3 joinpoints**
1980–1996 APC = −3.1*
1996–1999 APC = −4.8
1999–2011 APC = −0.4
2011–2021 APC = −3.9*

2. Akita Prefecture

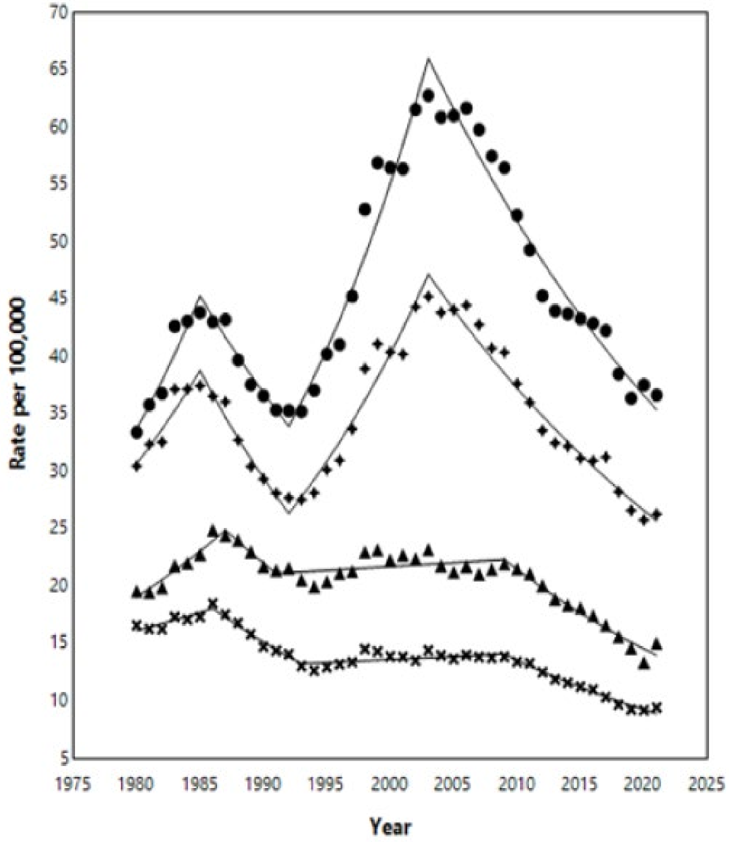

● **Male / All ages – 3 Joinpoints**
1980–1985 APC = 6.1*
1985–1992 APC = −4.0*
1992–2003 APC = 6.2*
2003–2021 APC = −3.4*

▲ **Female / All ages – 3 Joinpoints**
1980–1987 APC = 3.9*
1987–1991 APC = −4.0*
1991–2009 APC = 0.3
2009–2021 APC = −3.8*

✚ **Male / Age-standardized – 3 Joinpoints**
1980–1985 APC = 4.8*
1985–1992 APC = −5.4*
1992–2003 APC = 5.4*
2003–2021 APC = −3.3*

✕ **Female / Age-standardized – 3 Joinpoints**
1980–1986 APC = 1.9*
1986–1993 APC = −4.3*
1993–2009 APC = 0.4*
2009–2021 APC = −3.8*

3. Aomori Prefecture

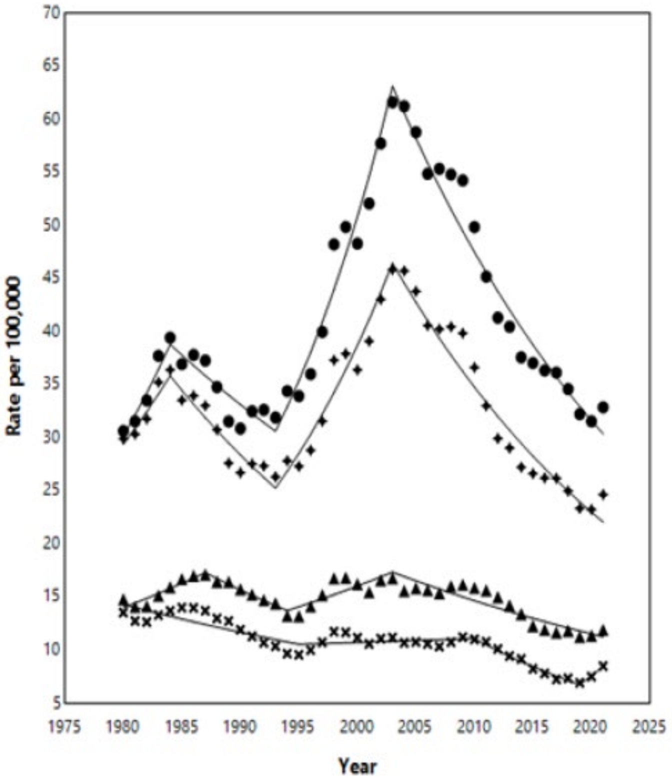

● **Male / All ages – 3 Joinpoints**
1980–1984 APC = 6.5*
1984–1993 APC = −2.6*
1993–2003 APC = 7.5*
2003–2021 APC = −4.0*

▲ **Female / All ages – 3 Joinpoints**
1980–1987 APC = 3.1*
1987–1994 APC = −3.3*
1994–2003 APC = 2.6*
2003–2021 APC = −2.4*

✚ **Male / Age-standardized – 3 Joinpoints**
1980–1984 APC = 5.1*
1984–1993 APC = −3.8*
1993–2003 APC = 6.3*
2003–2021 APC = −4.1*

✕ **Female / Age-standardized – 3 Joinpoints**
1980–1995 APC = −2.0*
1995–2010 APC = 0.3
2010–2019 APC = −5.4*
2019–2021 APC = 11.8

4. Chiba Prefecture

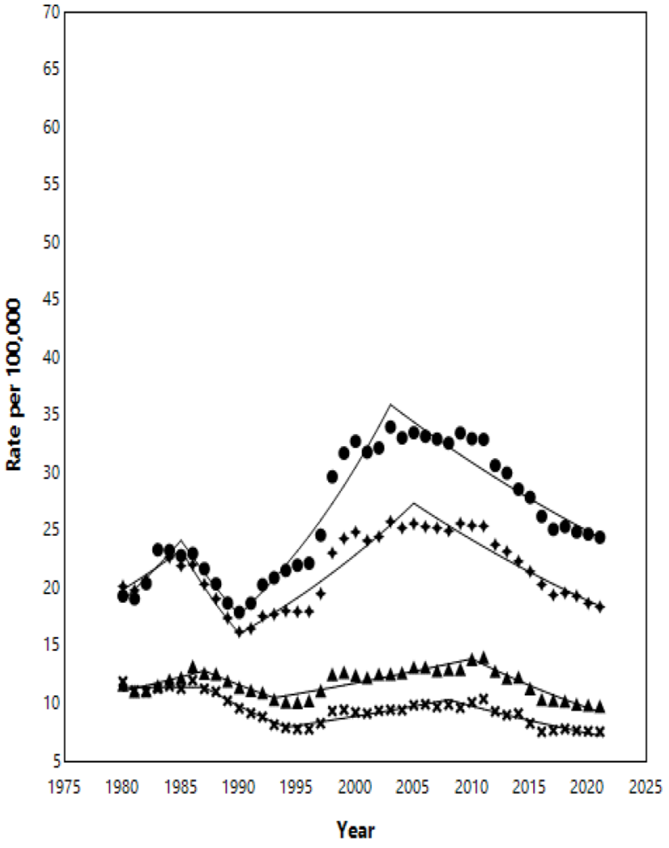

● **Male / All ages – 3 Joinpoints**
1980–1995 APC = −5.0*
1995–1999 APC = 8.8*
1999–2009 APC = 0.5
2009–2021 APC = −4.2*

▲ **Female / All ages – 3 Joinpoints**
1980–1993 APC = −2.3*
1993–1997 APC = −3.3*
1997–2004 APC = 1.7*
2004–2021 APC = −1.3*

✚ **Male / Age-standardized – 3 Joinpoints**
1980–1995 APC = −3.2*
1995–1999 APC = 7.2*
1999–2009 APC = 3.5*
2009–2021 APC = −4.4*

✕ **Female / Age-standardized – 3 Joinpoints**
1980–1993 APC = 0.0
1993–1997 APC = −5.1*
1997–2004 APC = 1.9*
2004–2021 APC = −2.7*

5. Ehime Prefecture

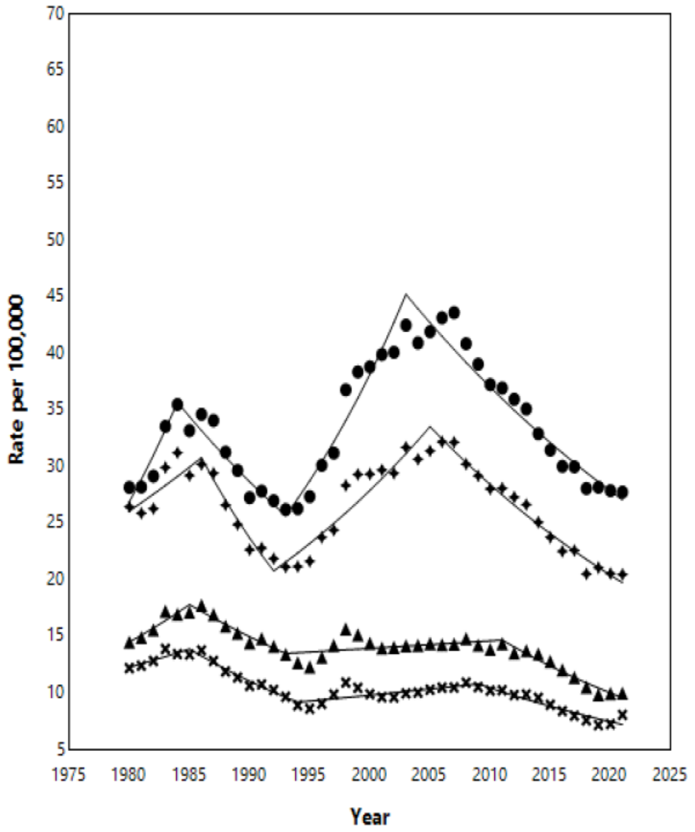

● Male / All ages – 3 Joinpoints
1980–1984 APC = 7.3*
1984–1993 APC = −3.6*
1993–2003 APC = 5.8*
2003–2021 APC = −2.8*

▲ Female / All ages – 3 Joinpoints
1980–1985 APC = 4.2*
1985–1993 APC = −3.4*
1993–2011 APC = 0.5*
2011–2021 APC = −4.2*

✚ Male / Age-standardized – 3 Joinpoints
1980–1986 APC = 2.8*
1986–1992 APC = −6.4*
1992–2005 APC = 3.8*
2005–2021 APC = −3.3*

✕ Female / Age-standardized – 3 Joinpoints
1980–1985 APC = 2.5
1985–1994 APC = −4.5*
1994–2009 APC = 1.1*
2009–2021 APC = −3.3*

6. Fukui Prefecture

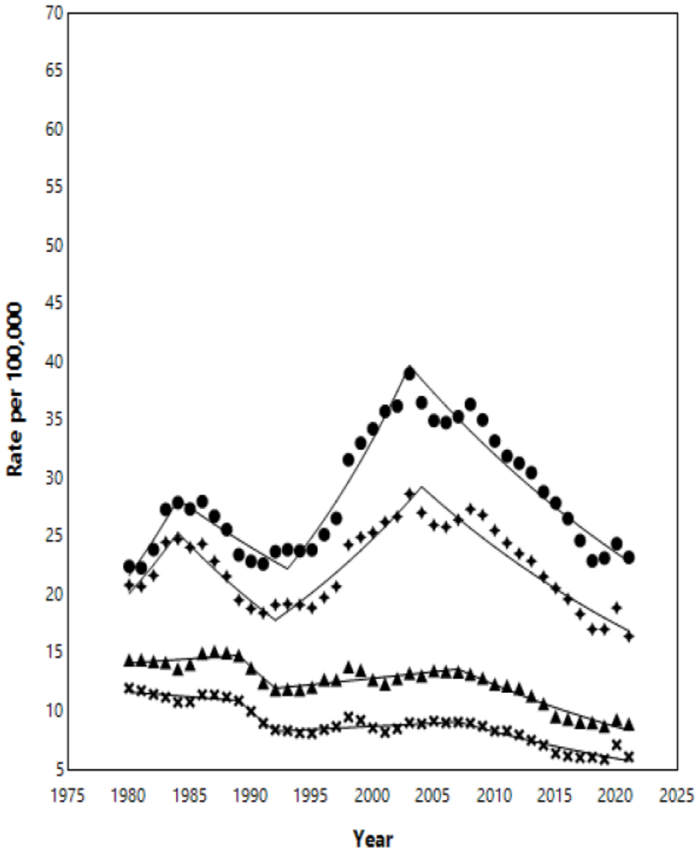

● Male / All ages – 3 Joinpoints
1980–1984 APC = 6.8*
1984–1993 APC = −2.6*
1993–2003 APC = 6.0*
2003–2021 APC = −3.0*

▲ Female / All ages – 3 Joinpoints
1980–1989 APC = 0.5
1989–1992 APC = −6.8*
1992–2007 APC = 0.9*
2007–2021 APC = −3.4*

✚ Male / Age-standardized – 3 Joinpoints
1980–1984 APC = 6.0*
1984–1992 APC = −4.3*
1992–2004 APC = 4.2*
2004–2021 APC = −3.2*

✕ Female / Age-standardized – 3 Joinpoints
1980–1989 APC = −0.7
1989–1992 APC = −6.8*
1992–2007 APC = 0.6
2007–2021 APC = −3.2*

7. Fukuoka Prefecture

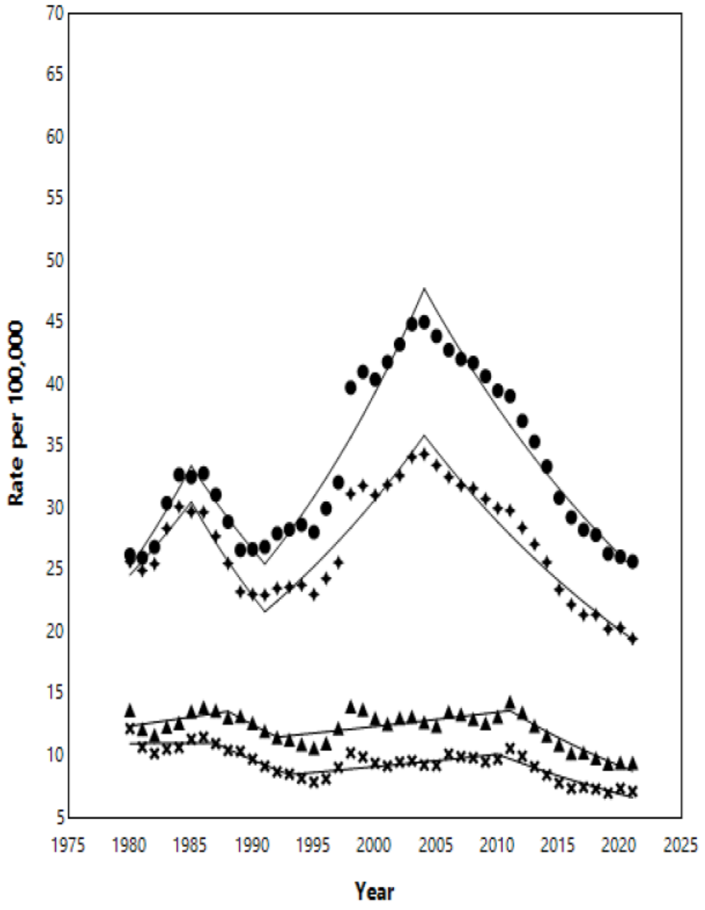

● **Male / All ages – 3 Joinpoints**
1980–1985 APC = 5.8*
1985–1991 APC = −4.4*
1991–2004 APC = 4.9*
2004–2021 APC = −3.7*

▲ **Female / All ages – 3 Joinpoints**
1980–1988 APC = 1.1
1988–1992 APC = −4.1
1992–2011 APC = 0.9*
2011–2021 APC = −4.3*

✚ **Male / Age-standardized – 3 Joinpoints**
1980–1985 APC = 4.4*
1985–1991 APC = −5.6*
1991–2004 APC = 4.0*
2004–2021 APC = −3.6*

✕ **Female / Age-standardized – 3 Joinpoints**
1980–1987 APC = 0.0
1987–1993 APC = −4.3*
1993–2010 APC = 1.1*
2010–2021 APC = −3.8*

8. Fukushima Prefecture

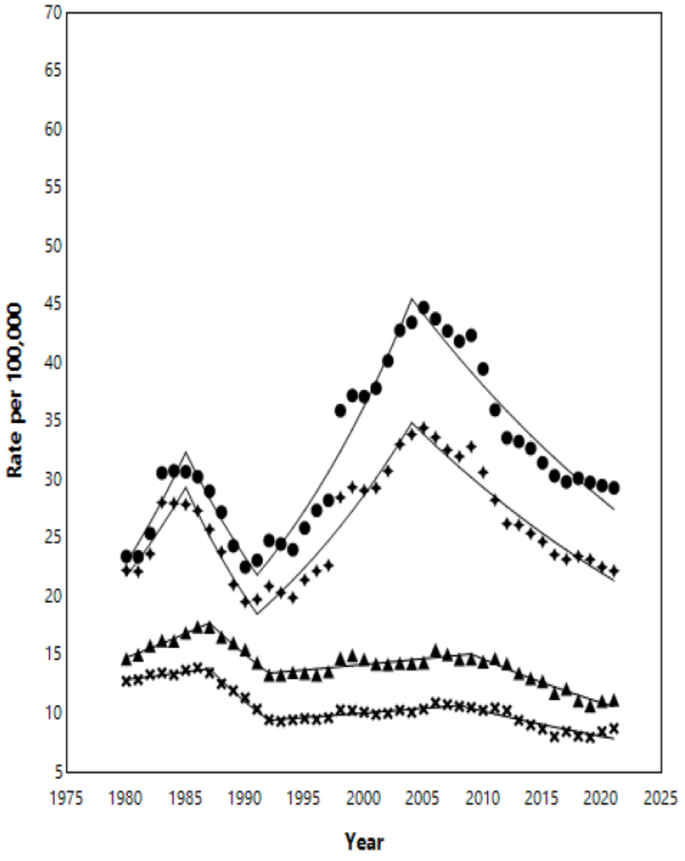

● **Male / All ages – 3 Joinpoints**
1980–1985 APC = 7.1*
1985–1991 APC = −6.3*
1991–2004 APC = 5.8*
2004–2021 APC = −2.9*

▲ **Female / All ages – 3 Joinpoints**
1980–1987 APC = 2.6*
1987–1992 APC = −5.4*
1992–2009 APC = 0.7*
2009–2021 APC = −2.9*

✚ **Male / Age-standardized – 3 Joinpoints**
1980–1985 APC = 6.2*
1985–1991 APC = −7.4*
1991–2004 APC = 5.0*
2004–2021 APC = −2.8*

✕ **Female / Age-standardized – 3 Joinpoints**
1980–1987 APC = 1.0
1987–1992 APC = −7.4*
1992–2008 APC = 0.8*
2008–2021 APC = −2.3*

9. Gifu Prefecture

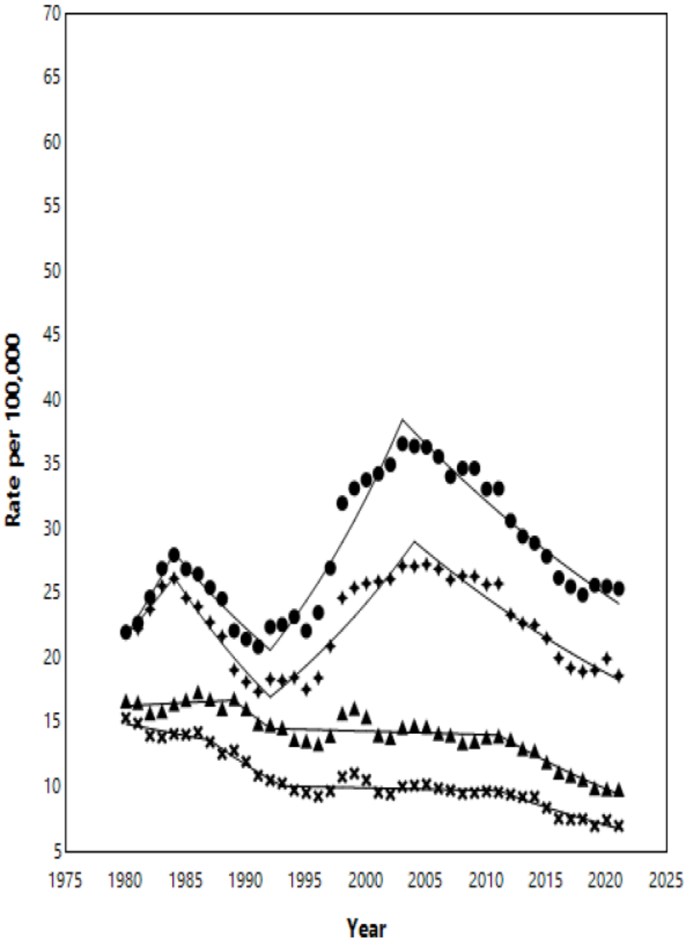

● Male / All ages – 3 Joinpoints
1980–1984 APC = 6.7*
1984–1992 APC = −3.8*
1992–2003 APC = 5.8*
2003–2021 APC = −2.5*

▲ Female / All ages – 3 Joinpoints
1980–1989 APC = 0.3
1989–1992 APC = −4.6
1992–2011 APC = −0.2
2011–2021 APC = −3.9*

✚ Male / Age-standardized – 3 Joinpoints
1980–1984 APC = 4.9*
1984–1992 APC = −5.3*
1992–2004 APC = 4.6*
2004–2021 APC = −2.7*

✕ Female / Age-standardized – 3 Joinpoints
1980–1987 APC = −1.3
1987–1993 APC = −4.9
1993–2011 APC = −0.2
2011–2021 APC = −3.5*

10. Gunma Prefecture

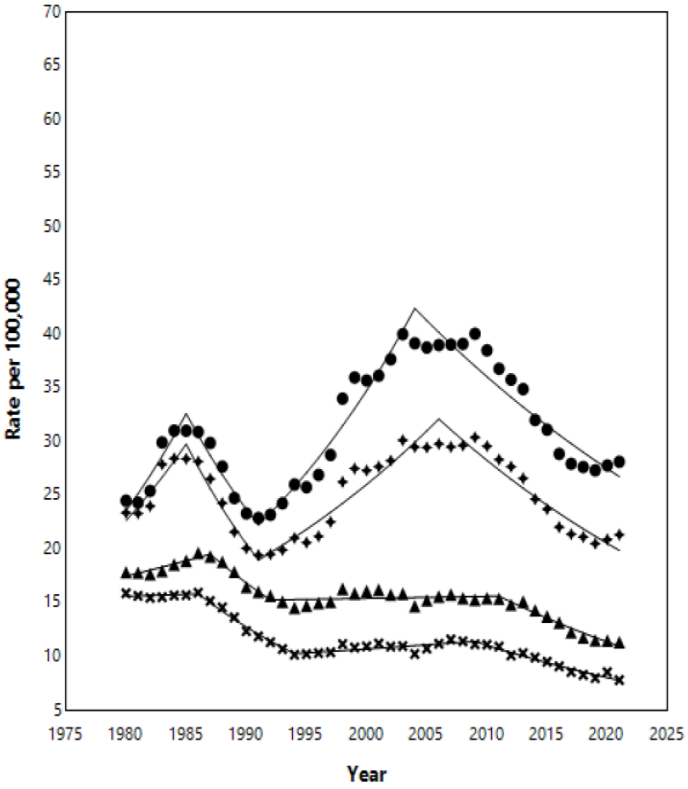

● Male / All ages – 3 Joinpoints
1980–1985 APC = 6.7*
1985–1991 APC = −6.2*
1991–2004 APC = 5.1*
2004–2021 APC = −2.7*

▲ Female / All ages – 3 Joinpoints
1980–1987 APC = 1.6*
1987–1992 APC = −4.8*
1992–2011 APC = 0.1
2011–2021 APC = −3.5*

✚ Male / Age-standardized – 3 Joinpoints
1980–1985 APC = 5.6*
1985–1991 APC = −7.2*
1991–2006 APC = 3.6*
2006–2021 APC = −3.2*

✕ Female / Age-standardized – 3 Joinpoints
1980–1986 APC = 0.2
1986–1994 APC = −5.3*
1994–2009 APC = 0.7*
2009–2021 APC = −3.2*

11. Hiroshima Prefecture

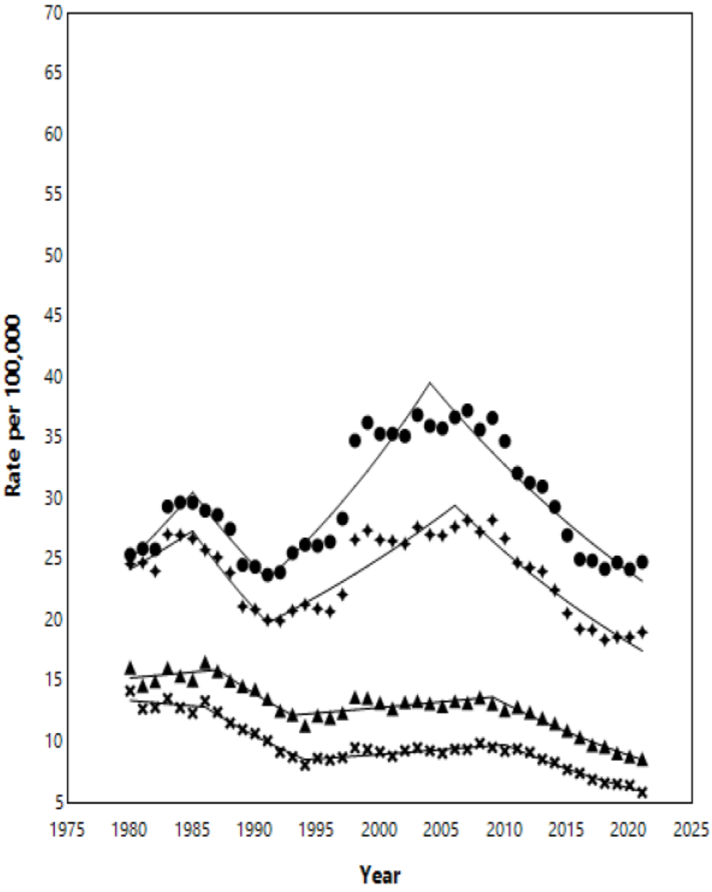

● Male / All ages – 3 Joinpoints
1980–1985 APC = 4.1*
1985–1991 APC = −4.4*
1991–2004 APC = 4.1*
2004–2021 APC = −3.1*

▲ Female / All ages – 3 Joinpoints
1980–1987 APC = 0.6
1987–1993 APC = −4.4*
1993–2009 APC = 0.7*
2009–2021 APC = −3.9*

✚ Male / Age-standardized – 3 Joinpoints
1980–1985 APC = 2.5
1985–1991 APC = −5.3*
1991–2006 APC = 2.7*
2006–2021 APC = −3.4*

✕ Female / Age-standardized – 3 Joinpoints
1980–1986 APC = −0.7
1986–1994 APC = −5.0*
1994–2010 APC = 0.8*
2010–2021 APC = −4.5*

12. Hokkaido Prefecture

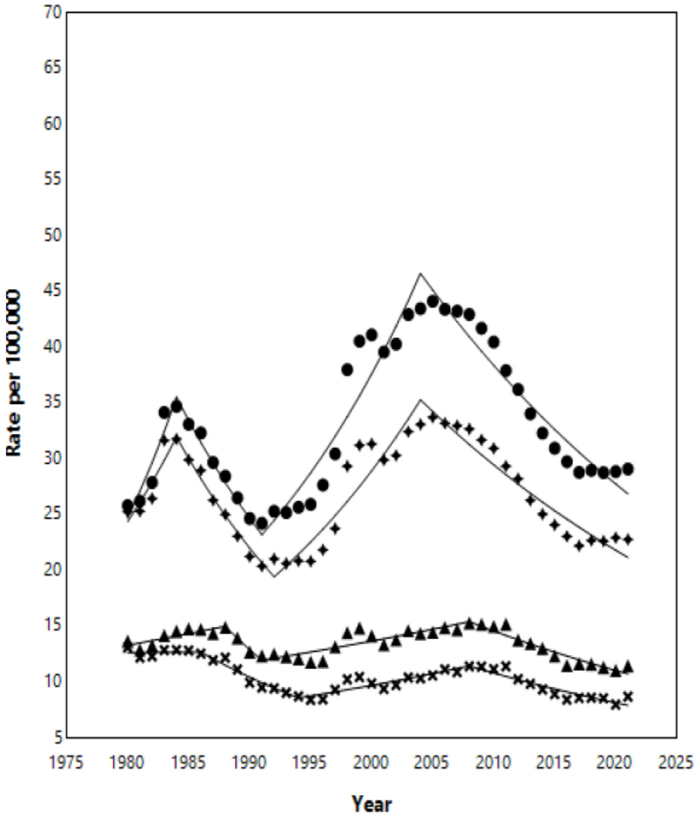

● Male / All ages – 3 Joinpoints
1980–1984 APC = 9.5*
1984–1991 APC = −5.9*
1991–2004 APC = 5.5*
2004–2021 APC = −3.2*

▲ Female / All ages – 3 Joinpoints
1980–1988 APC = 1.5*
1988–1991 APC = −7.3*
1991–2008 APC = 1.5*
2008–2021 APC = −2.8*

✚ Male / Age-standardized – 3 Joinpoints
1980–1984 APC = 7.0*
1984–1992 APC = −6.0*
1992–2004 APC = 5.1*
2004–2021 APC = −3.0*

✕ Female / Age-standardized – 3 Joinpoints
1980–1986 APC = 0.1
1986–1994 APC = −4.8*
1994–2008 APC = 2.1*
2008–2021 APC = −2.8*

13. Hyogo Prefecture

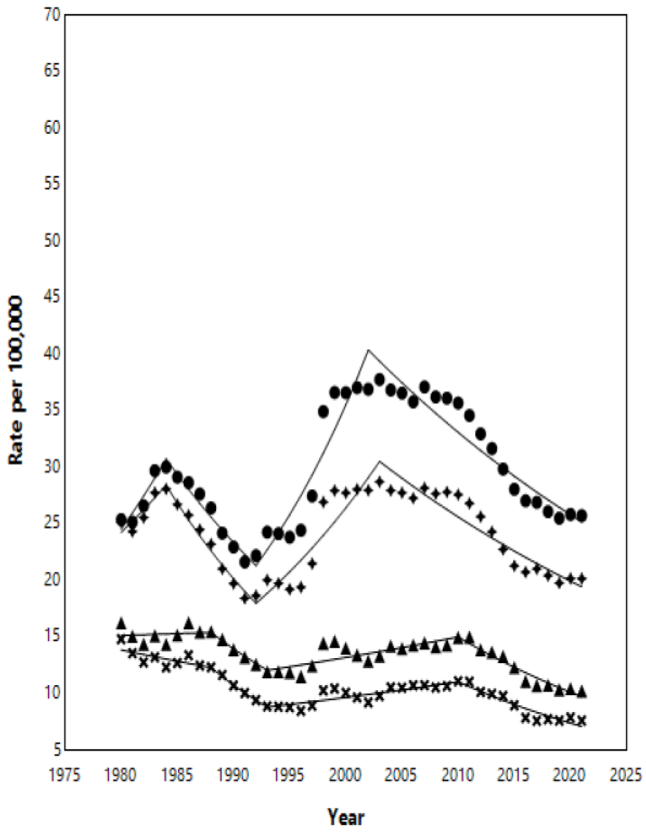

● **Male / All ages – 3 Joinpoints**
1980–1984 APC = 5.8*
1984–1992 APC = −4.5*
1992–2002 APC = 6.6*
2002–2021 APC = −2.5*

▲ **Female / All ages – 3 Joinpoints**
1980–1988 APC = 0.2
1988–1993 APC = −4.8*
1993–2010 APC = 1.3*
2010–2021 APC = −3.9*

✚ **Male / Age-standardized – 3 Joinpoints**
1980–1984 APC = 4.0
1984–1992 APC = −5.6*
1992–2003 APC = 4.9*
2003–2021 APC = −2.5*

✕ **Female / Age-standardized – 3 Joinpoints**
1980–1988 APC = −1.5
1988–1993 APC = −6.5*
1993–2010 APC = 1.4*
2010–2021 APC = −4.0*

14. Ibaraki Prefecture

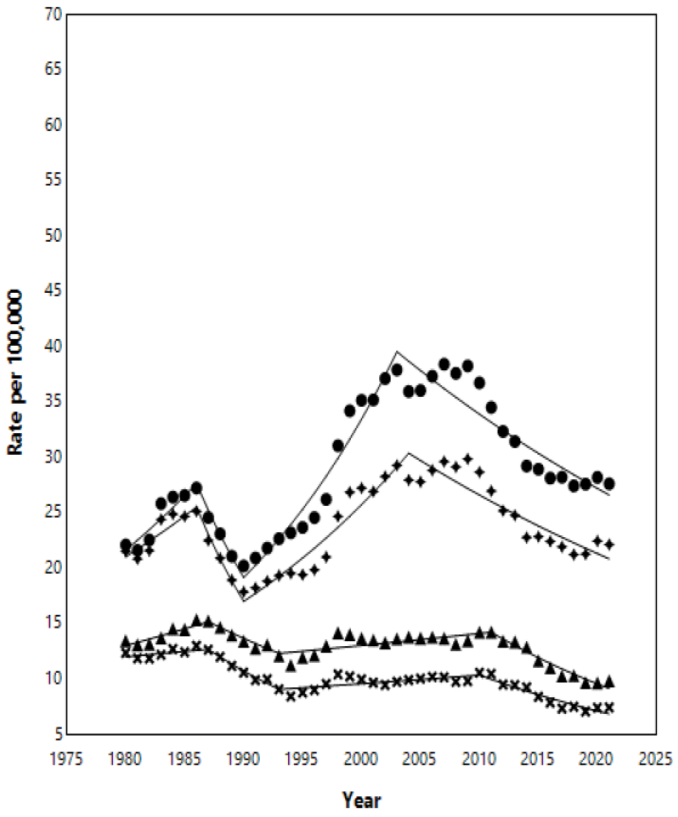

● **Male / All ages – 3 Joinpoints**
1980–1986 APC = 4.2*
1986–1990 APC = −8.8*
1990–2003 APC = 5.7*
2003–2021 APC = −2.2*

▲ **Female / All ages – 3 Joinpoints**
1980–1987 APC = 2.2*
1987–1993 APC = −3.4*
1993–2011 APC = 0.8*
2011–2021 APC = −4.3*

✚ **Male / Age-standardized – 3 Joinpoints**
1980–1986 APC = 3.3*
1986–1990 APC = −9.7*
1990–2004 APC = 4.2*
2004–2021 APC = −2.2*

✕ **Female / Age-standardized – 3 Joinpoints**
1980–1987 APC = 0.8
1987–1993 APC = −5.5*
1993–2010 APC = 0.8*
2010–2021 APC = −3.7*

15. Ishikawa Prefecture

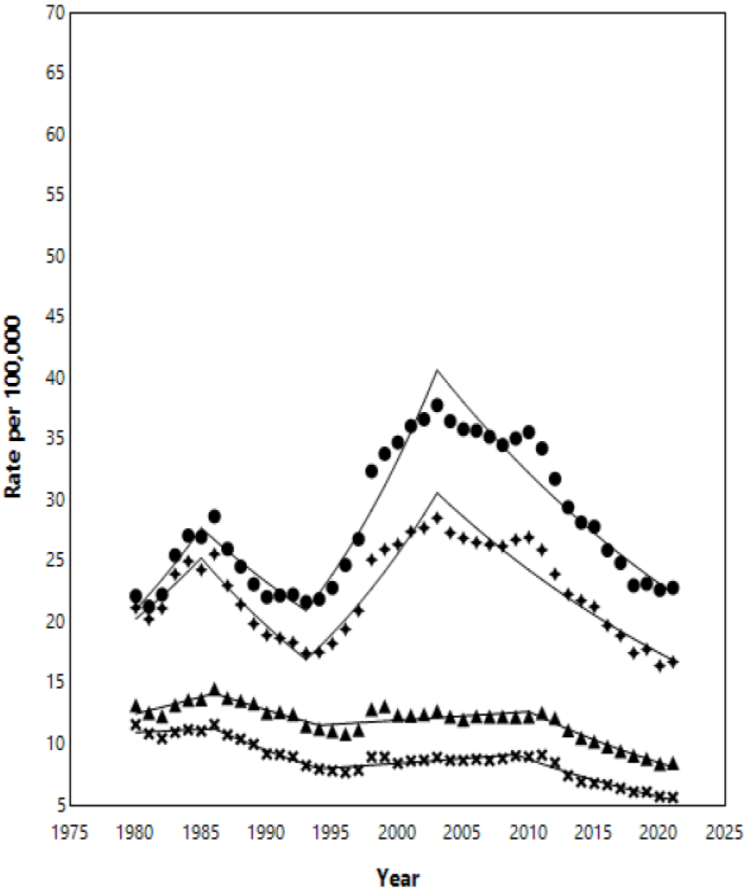

● **Male / All ages – 3 Joinpoints**
1980–1985 APC = 5.6*
1985–1993 APC = −3.4*
1993–2003 APC = 6.9*
2003–2021 APC = −3.3*

▲ **Female / All ages – 3 Joinpoints**
1980–1986 APC = 2.1*
1986–1994 APC = −2.5*
1994–2010 APC = 0.6*
2010–2021 APC = −4.0*

✚ **Male / Age-standardized – 3 Joinpoints**
1980–1985 APC = 4.5*
1985–1993 APC = −4.9*
1993–2003 APC = 6.1*
2003–2021 APC = −3.3*

✕ **Female / Age-standardized – 3 Joinpoints**
1980–1986 APC = 0.4
1986–1994 APC = −4.2*
1994–2009 APC = 0.9*
2009–2021 APC = −4.2*

16. Iwate Prefecture

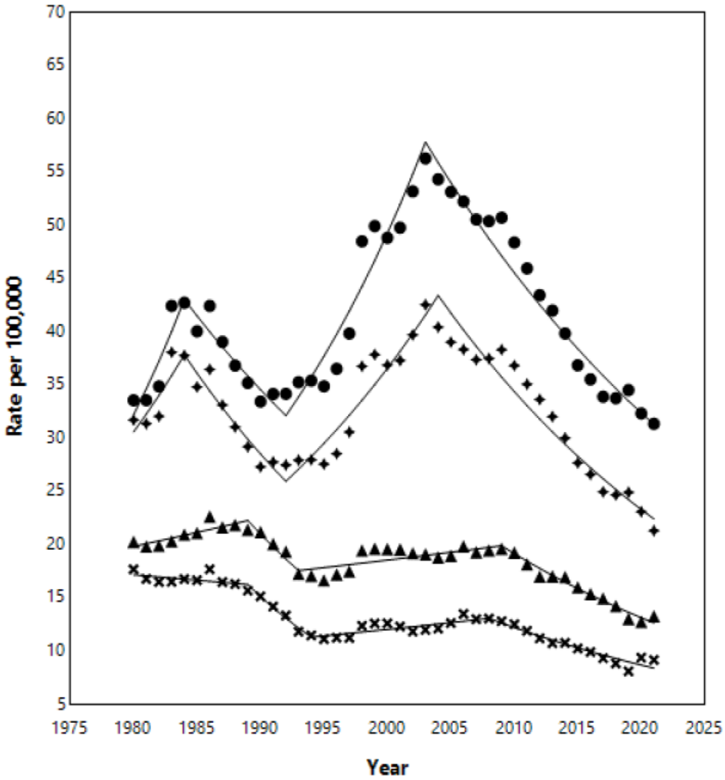

● **Male / All ages – 3 Joinpoints**
1980–1984 APC = 7.5*
1984–1992 APC = −3.6*
1992–2003 APC = 5.5*
2003–2021 APC = −3.4*

▲ **Female / All ages – 3 Joinpoints**
1980–1989 APC = 1.3*
1989–1993 APC = −5.8*
1993–2009 APC = 0.8*
2009–2021 APC = −3.7*

✚ **Male / Age-standardized – 3 Joinpoints**
1980–1984 APC = 5.5*
1984–1992 APC = −4.6*
1992–2004 APC = 4.4*
2004–2021 APC = −3.8*

✕ **Female / Age-standardized – 3 Joinpoints**
1980–1989 APC = −0.6
1989–1994 APC = −7.0*
1994–2008 APC = 1.0*
2008–2021 APC = −3.4*

17. Kagawa Prefecture

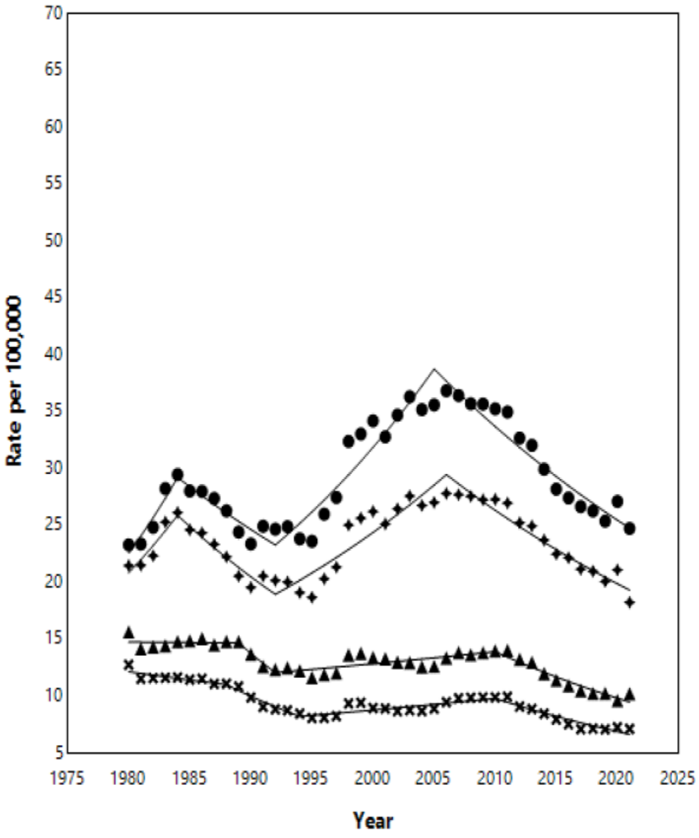

● Male / All ages – 3 Joinpoints
1980–1984 APC = 6.7*
1984–1992 APC = −2.8*
1992–2005 APC = 4.0*
2005–2021 APC = −2.8*

▲ Female / All ages – 3 Joinpoints
1980–1989 APC = −0.1
1989–1992 APC = −6.4*
1992–2010 APC = 0.8*
2010–2021 APC = −3.4*

✚ Male / Age-standardized – 3 Joinpoints
1980–1984 APC = 5.6*
1984–1992 APC = −3.8*
1992–2006 APC = 3.2*
2006–2021 APC = −2.8*

✕ Female / Age-standardized – 3 Joinpoints
1980–1988 APC = −1.2
1988–1994 APC = −4.8
1994–2010 APC = 1.1
2010–2021 APC = −3.5*

18. Kagoshima Prefecture

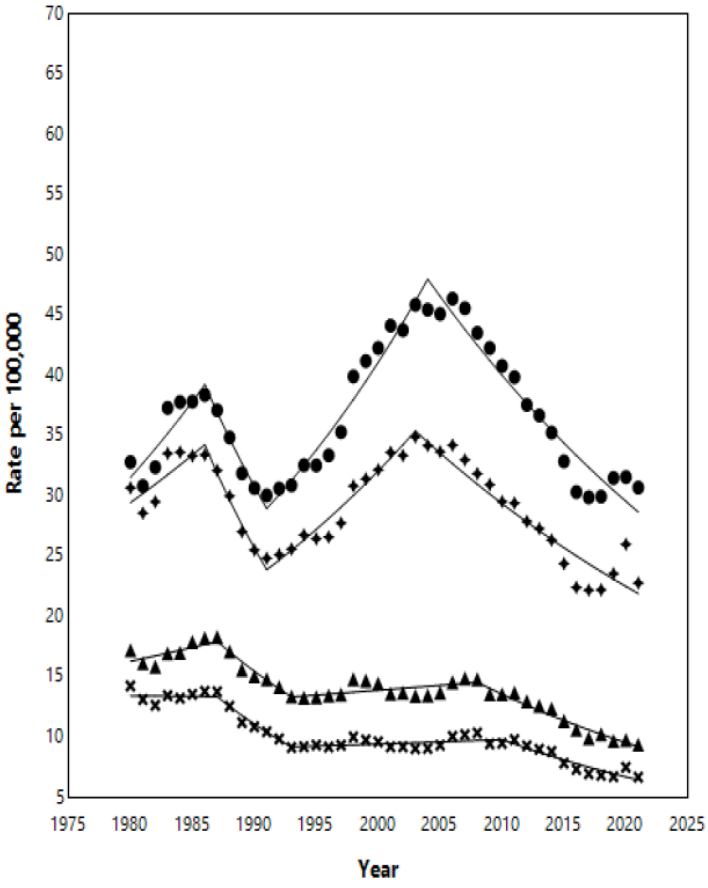

● Male / All ages – 3 Joinpoints
1980–1986 APC = 3.7*
1986–1991 APC = −5.9*
1991–2004 APC = 4.0*
2004–2021 APC = −3.0*

▲ Female / All ages – 3 Joinpoints
1980–1987 APC = 1.4*
1987–1993 APC = −4.8*
1993–2008 APC = 0.6*
2008–2021 APC = −3.5*

✚ Male / Age-standardized – 3 Joinpoints
1980–1986 APC = 2.6*
1986–1991 APC = −7.0*
1991–2003 APC = 3.3*
2003–2021 APC = −2.6*

✕ Female / Age-standardized – 3 Joinpoints
1980–1987 APC = −0.1
1987–1993 APC = −6.1*
1993–2010 APC = 0.4
2010–2021 APC = −3.7*

19. Kanagawa Prefecture

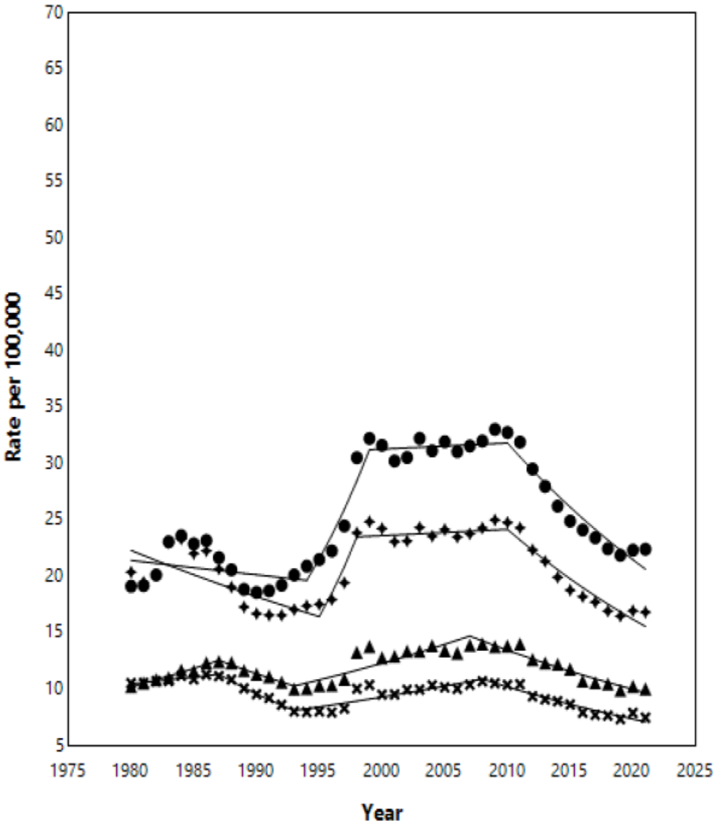

● Male / All ages – 3 Joinpoints
1980–1994 APC = −0.6
1994–1999 APC = 9.7*
1999–2010 APC = 0.2
2010–2021 APC = −3.9*

▲ Female / All ages – 3 Joinpoints
1980–1987 APC = 3.0*
1987–1993 APC = −3.3*
1993–2007 APC = 2.6*
2007–2021 APC = −2.9*

✚ Male / Age-standardized – 3 Joinpoints
1980–1995 APC = −2.0*
1995–1998 APC = 12.7*
1998–2010 APC = 0.2
2010–2021 APC = −3.9*

✕ Female / Age-standardized – 3 Joinpoints
1980–1987 APC = 1.1
1987–1993 APC = −5.4*
1993–2008 APC = 2.0*
2008–2021 APC = −3.2*

20. Kochi Prefecture

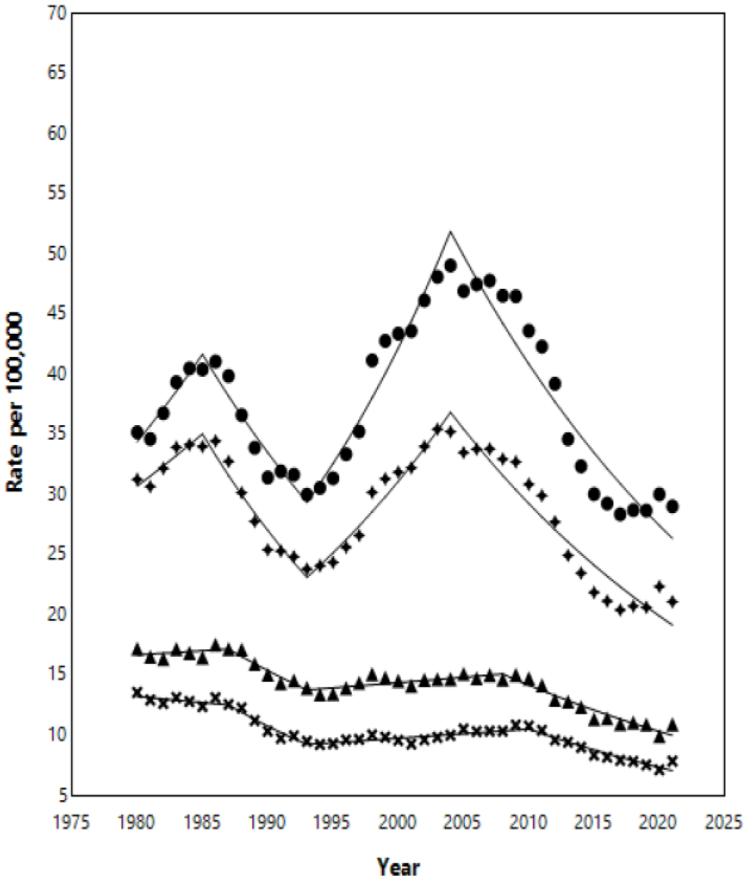

● Male / All ages – 3 Joinpoints
1980–1985 APC = 3.9*
1985–1993 APC = −4.3*
1993–2004 APC = 5.3*
2004–2021 APC = −3.9*

▲ Female / All ages – 3 Joinpoints
1980–1987 APC = 0.4
1987–1993 APC = −3.6*
1993–2008 APC = 0.6*
2008–2021 APC = −3.1*

✚ Male / Age-standardized – 3 Joinpoints
1980–1985 APC = 2.7
1985–1993 APC = −5.1*
1993–2004 APC = 4.3*
2004–2021 APC = −3.8*

✕ Female / Age-standardized – 3 Joinpoints
1980–1987 APC = −0.8
1987–1993 APC = −4.9*
1993–2010 APC = 0.8*
2010–2021 APC = −3.6*

21. Kumamoto Prefecture

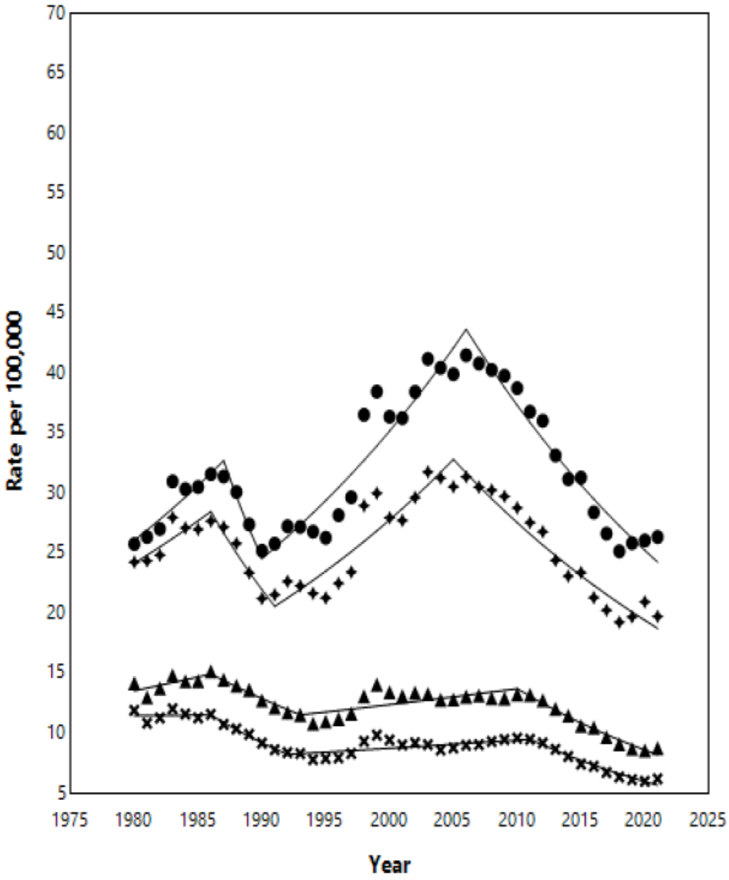

● Male / All ages – 3 Joinpoints
1980–1987 APC = 3.3*
1987–1990 APC = −9.1*
1990–2006 APC = 3.7*
2006–2021 APC = −3.8*

▲ Female / All ages – 3 Joinpoints
1980–1986 APC = 1.6
1986–1993 APC = −3.6*
1993–2010 APC = 1.0*
2010–2021 APC = −4.6*

✚ Male / Age-standardized – 3 Joinpoints
1980–1986 APC = 2.8*
1986–1991 APC = −6.3*
1991–2005 APC = 3.4*
2005–2021 APC = −3.4*

✕ Female / Age-standardized – 3 Joinpoints
1980–1986 APC = 0.1
1986–1992 APC = −5.4*
1992–2011 APC = 0.8*
2011–2021 APC = −5.1*

22. Kyoto Prefecture

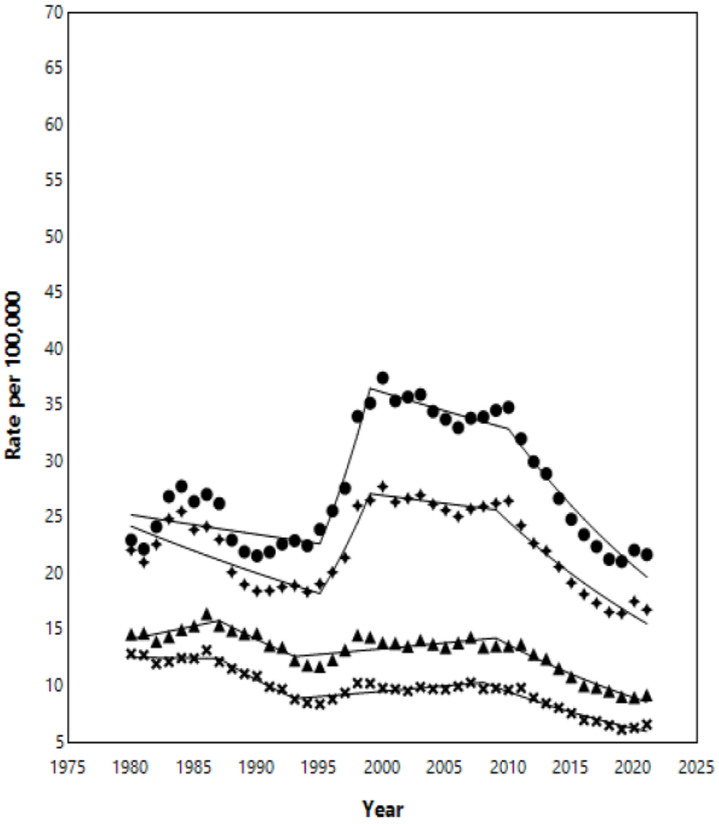

● Male / All ages – 3 Joinpoints
1980–1995 APC = −0.7
1995–1999 APC = 12.6*
1999–2010 APC = −0.9
2010–2021 APC = −4.6*

▲ Female / All ages – 3 Joinpoints
1980–1987 APC = 1.6*
1987–1993 APC = −3.7*
1993–2009 APC = 0.8*
2009–2021 APC = −4.2*

✚ Male / Age-standardized – 3 Joinpoints
1980–1995 APC = −1.9*
1995–1999 APC = 10.5*
1999–2009 APC = −0.5
2009–2021 APC = −4.1*

✕ Female / Age-standardized – 3 Joinpoints
1980–1987 APC = −0.1
1987–1993 APC = −5.5*
1993–2008 APC = 1.0*
2008–2021 APC = −4.1*

23. Mie Prefecture

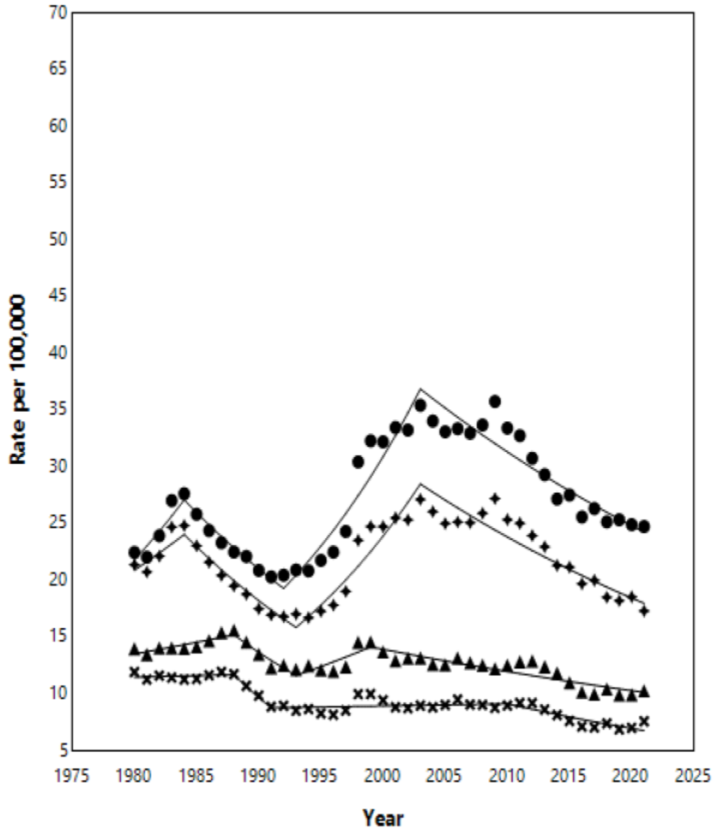

● Male / All ages – 3 Joinpoints
1980–1984 APC = 5.6*
1984–1992 APC = −4.2*
1992–2003 APC = 6.1*
2003–2021 APC = −2.3*

▲ Female / All ages – 3 Joinpoints
1980–1988 APC = 1.5*
1988–1993 APC = −5.2*
1993–1999 APC = 3.3*
1999–2021 APC = −1.5*

✚ Male / Age-standardized – 3 Joinpoints
1980–1984 APC = 3.6
1984–1993 APC = −4.5*
1993–2003 APC = 6.1*
2003–2021 APC = −2.5*

✕ Female / Age-standardized – 3 Joinpoints
1980–1988 APC = 0.2
1988–1991 APC = −9.2*
1991–2010 APC = 0.2
2010–2021 APC = −2.7*

24. Miyagi Prefecture

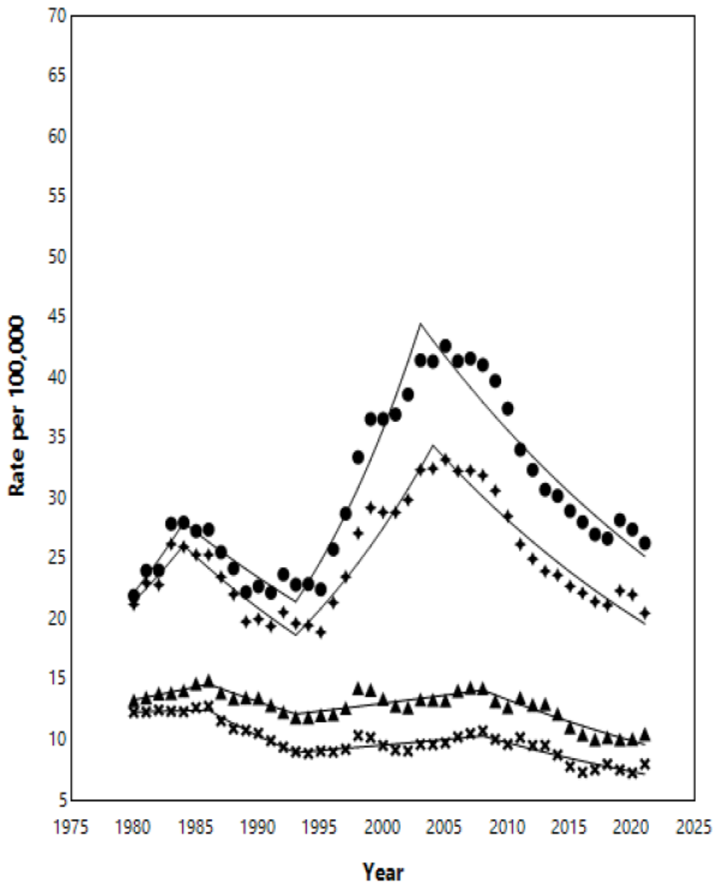

● Male / All ages – 3 Joinpoints
1980–1984 APC = 6.0*
1984–1993 APC = −2.9*
1993–2003 APC = 7.6*
2003–2021 APC = −3.1*

▲ Female / All ages – 3 Joinpoints
1980–1986 APC = 1.6
1986–1993 APC = −2.6
1993–2008 APC = 1.0*
2008–2021 APC = −2.9*

✚ Male / Age-standardized – 3 Joinpoints
1980–1984 APC = 5.0*
1984–1993 APC = −3.7*
1993–2004 APC = 5.7*
2004–2021 APC = −3.3*

✕ Female / Age-standardized – 3 Joinpoints
1980–1986 APC = 0.2
1986–1993 APC = −4.6*
1993–2008 APC = 1.0*
2008–2021 APC = −2.8*

25. Miyazaki Prefecture

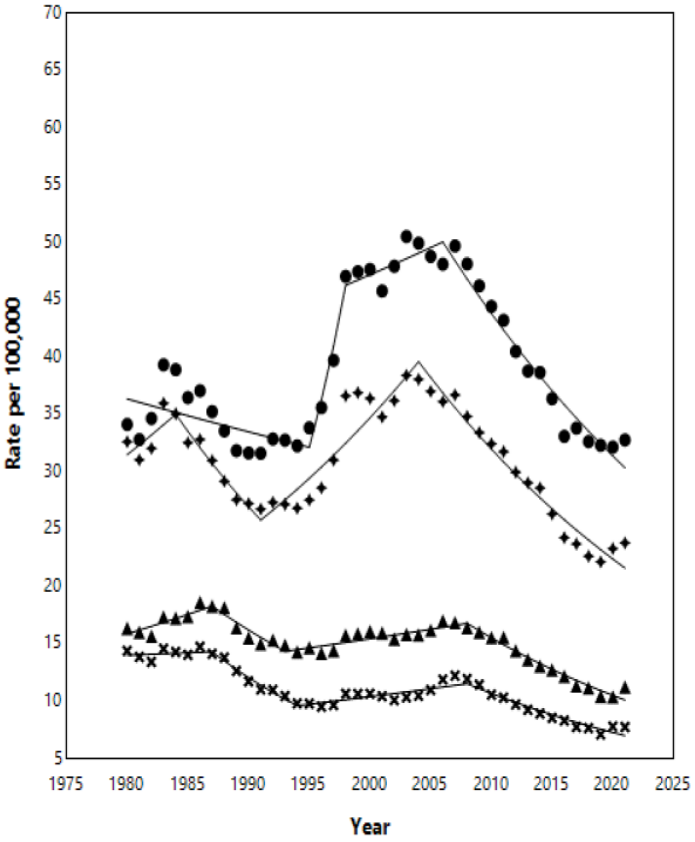

● Male / All ages – 3 Joinpoints
1980–1995 APC = −0.8*
1995–1998 APC = 12.9*
1998–2006 APC = 1.0
2006–2021 APC = −3.3*

▲ Female / All ages – 3 Joinpoints
1980–1987 APC = 2.0*
1987–1993 APC = −4.0*
1993–2008 APC = 1.1*
2008–2021 APC = −3.8*

✚ Male / Age-standardized – 3 Joinpoints
1980–1984 APC = 2.7
1984–1991 APC = −4.3
1991–2004 APC = 3.4*
2004–2021 APC = −3.5*

✕ Female / Age-standardized – 3 Joinpoints
1980–1987 APC = 0.3
1987–1994 APC = −5.5*
1994–2008 APC = 1.3*
2008–2021 APC = −3.8*

26. Nagano Prefecture

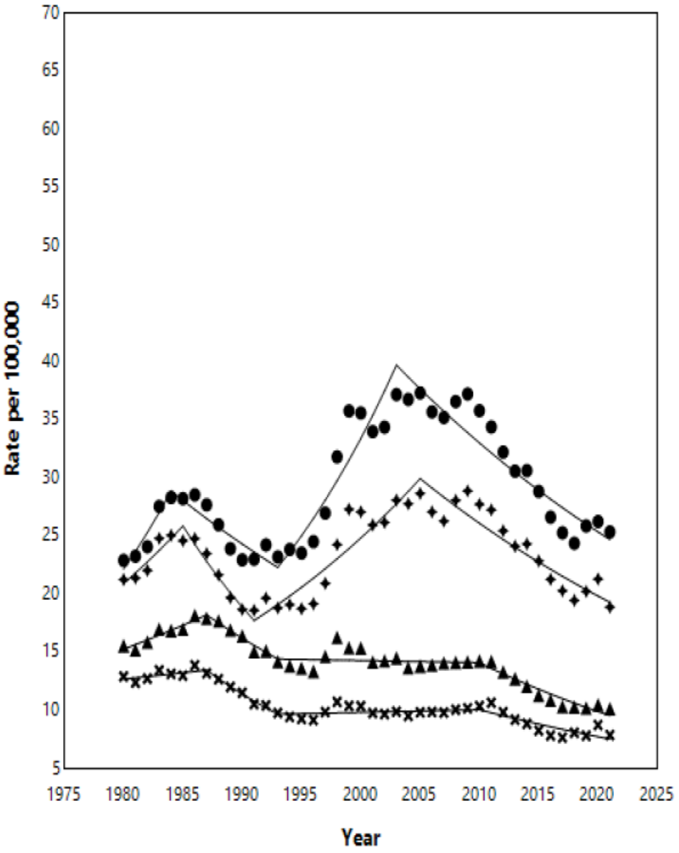

● Male / All ages – 3 Joinpoints
1980–1984 APC = 6.9*
1984–1993 APC = −2.8*
1993–2003 APC = 5.9*
2003–2021 APC = −2.6*

▲ Female / All ages – 3 Joinpoints
1980–1987 APC = 2.6*
1987–1993 APC = −3.8*
1993–2010 APC = −0.1
2010–2021 APC = −3.5*

✚ Male / Age-standardized – 3 Joinpoints
1980–1985 APC = 4.4*
1985–1991 APC = −6.1*
1991–2005 APC = 3.8*
2005–2021 APC = −2.7*

✕ Female / Age-standardized – 3 Joinpoints
1980–1987 APC = 0.8
1987–1993 APC = −5.4*
1993–2010 APC = 0.2
2010–2021 APC = −2.6*

27. Nagasaki Prefecture

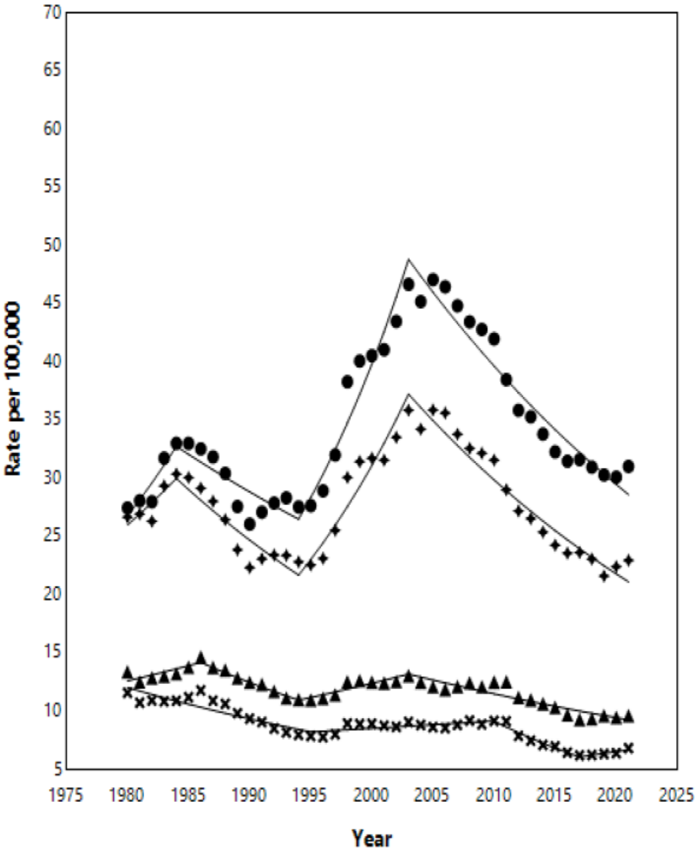

● Male / All ages – 3 Joinpoints
1980–1984 APC = 5.2*
1984–1994 APC = −2.1*
1994–2003 APC = 7.0*
2003–2021 APC = −2.9*

▲ Female / All ages – 3 Joinpoints
1980–1986 APC = 2.0*
1986–1994 APC = −3.2*
1994–2003 APC = 2.1*
2003–2021 APC = −2.0*

✚ Male / Age-standardized – 3 Joinpoints
1980–1984 APC = 3.7
1984–1994 APC = −3.2*
1994–2003 APC = 6.2*
2003–2021 APC = −3.1*

✕ Female / Age-standardized – 3 Joinpoints
1980–1995 APC = −2.5*
1995–2010 APC = 0.7*
2010–2017 APC = −5.7*
2017–2021 APC = 2.4

28. Nara Prefecture

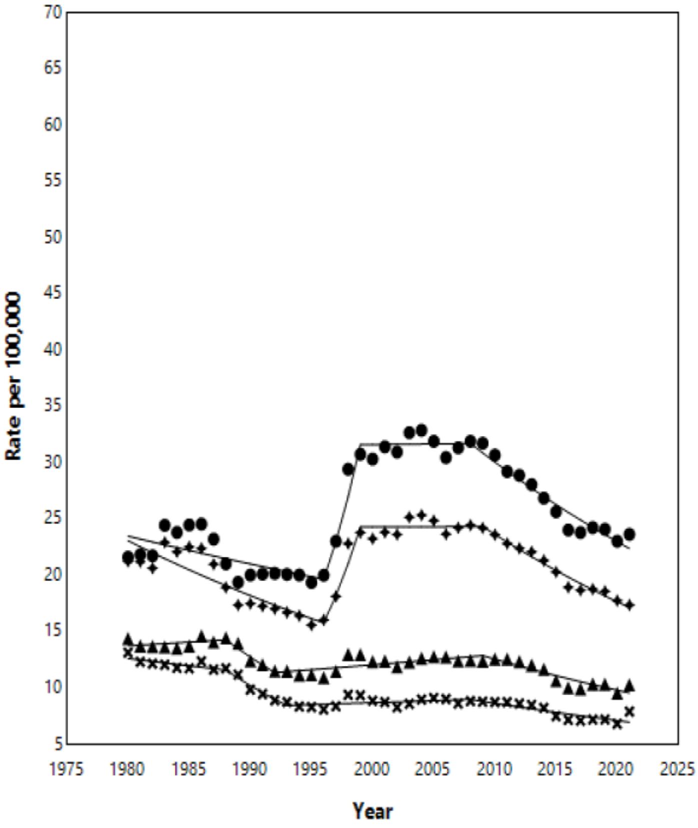

● Male / All ages – 3 Joinpoints
1980–1996 APC = −1.1*
1996–1999 APC = 17.2*
1999–2008 APC = 0.0
2008–2021 APC = −2.6*

▲ Female / All ages – 3 Joinpoints
1980–1988 APC = 0.5
1988–1992 APC = −5.5*
1992–2009 APC = 0.7*
2009–2021 APC = −2.5*

✚ Male / Age-standardized – 3 Joinpoints
1980–1996 APC = −2.4*
1996–1999 APC = 15.5*
1999–2009 APC = 0.0
2009–2021 APC = −2.9*

✕ Female / Age-standardized – 3 Joinpoints
1980–1988 APC = −1.1
1988–1993 APC = −6.2*
1993–2008 APC = 0.4
2008–2021 APC = −2.0*

29. Niigata Prefecture

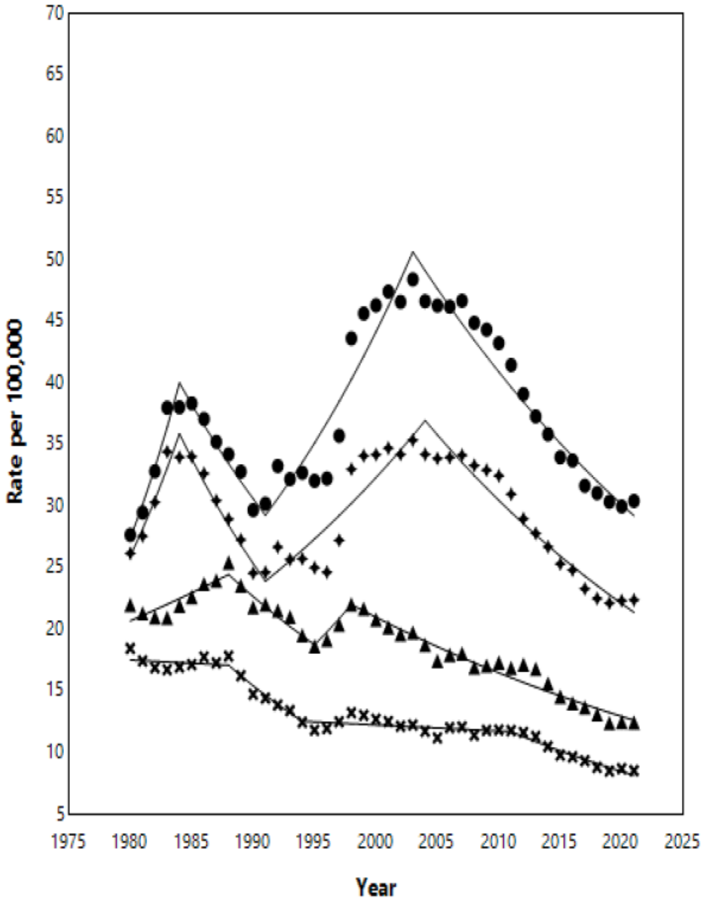

● Male / All ages – 3 Joinpoints
1980–1984 APC = 9.9*
1984–1991 APC = −4.4*
1991–2003 APC = 4.7*
2003–2021 APC = −3.0*

▲ Female / All ages – 3 Joinpoints
1980–1988 APC = 2.1*
1988–1995 APC = −3.7*
1995–1998 APC = 5.5*
1998–2021 APC = −2.4*

✚ Male / Age-standardized – 3 Joinpoints
1980–1984 APC = 8.4*
1984–1991 APC = −5.6*
1991–2004 APC = 3.4*
2004–2021 APC = −3.2*

✕ Female / Age-standardized – 3 Joinpoints
1980–1988 APC = −0.3
1988–1994 APC = −5.0*
1994–2011 APC = −0.4
2011–2021 APC = −3.5*

30. Oita Prefecture

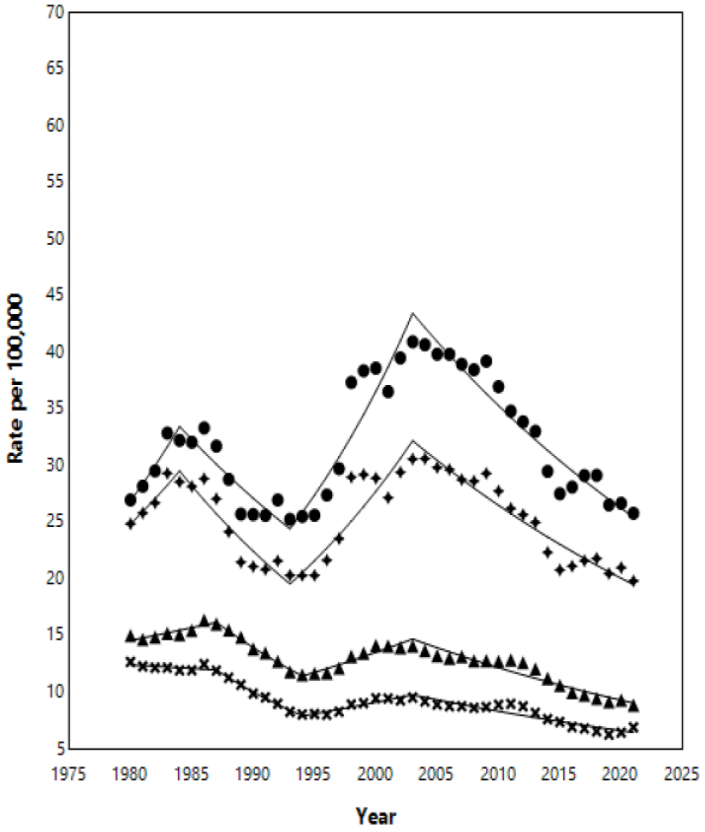

● Male / All ages – 3 Joinpoints
1980–1984 APC = 5.6*
1984–1993 APC = −3.4*
1993–2003 APC = 5.9*
2003–2021 APC = −2.9*

▲ Female / All ages – 3 Joinpoints
1980–1987 APC = 1.5*
1987–1994 APC = −4.8*
1994–2003 APC = 2.8*
2003–2021 APC = −2.7*

✚ Male / Age-standardized – 3 Joinpoints
1980–1984 APC = 4.4*
1984–1993 APC = −4.5*
1993–2003 APC = 5.1*
2003–2021 APC = −2.8*

✕ Female / Age-standardized – 3 Joinpoints
1980–1987 APC = −0.5
1987–1994 APC = −5.8*
1994–2003 APC = 2.4*
2003–2021 APC = −2.3*

31. Okayama Prefecture

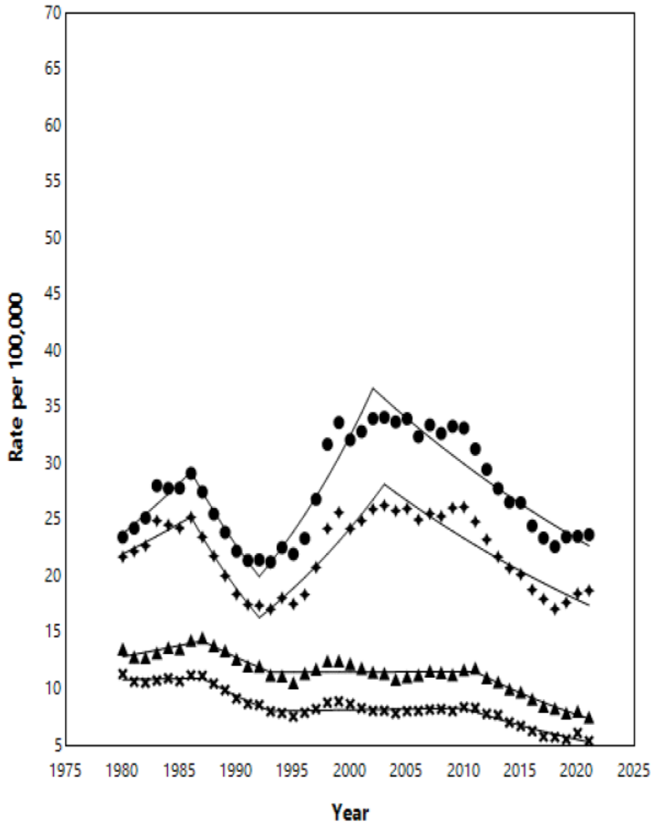

● Male / All ages – 3 Joinpoints
1980–1986 APC = 3.5*
1986–1992 APC = −6.2*
1992–2002 APC = 6.3*
2002–2021 APC = −2.5*

▲ Female / All ages – 3 Joinpoints
1980–1987 APC = 1.5*
1987–1993 APC = −3.6*
1993–2011 APC = 0.0
2011–2021 APC = −4.4*

✚ Male / Age-standardized – 3 Joinpoints
1980–1986 APC = 2.4*
1986–1992 APC = −7.0*
1992–2003 APC = 5.1*
2003–2021 APC = −2.6*

✕ Female / Age-standardized – 3 Joinpoints
1980–1987 APC = 0.2
1987–1993 APC = −5.0*
1993–2010 APC = 0.2
2010–2021 APC = −4.0*

32. Okinawa Prefecture

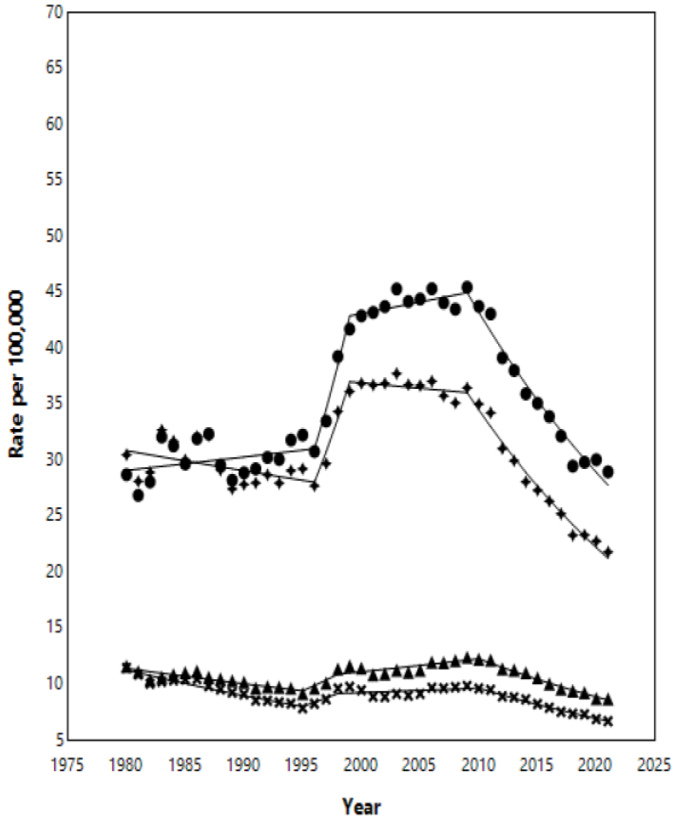

● Male / All ages – 3 Joinpoints
1980–1996 APC = 0.4
1996–1999 APC = 11.4*
1999–2009 APC = 0.5
2009–2021 APC = −3.0*

▲ Female / All ages – 3 Joinpoints
1980–1995 APC = −1.3*
1995–1998 APC = 5.0*
1998–2010 APC = 1.0
2010–2021 APC = −3.2*

✚ Male / Age-standardized – 3 Joinpoints
1980–1996 APC = −0.6*
1996–1999 APC = 9.7*
1999–2009 APC = −0.3
2009–2021 APC = −4.3*

✕ Female / Age-standardized – 3 Joinpoints
1980–1995 APC = −2.3*
1995–1998 APC = 4.6
1998–2010 APC = 0.4
2010–2021 APC = −3.3*

33. Osaka Prefecture

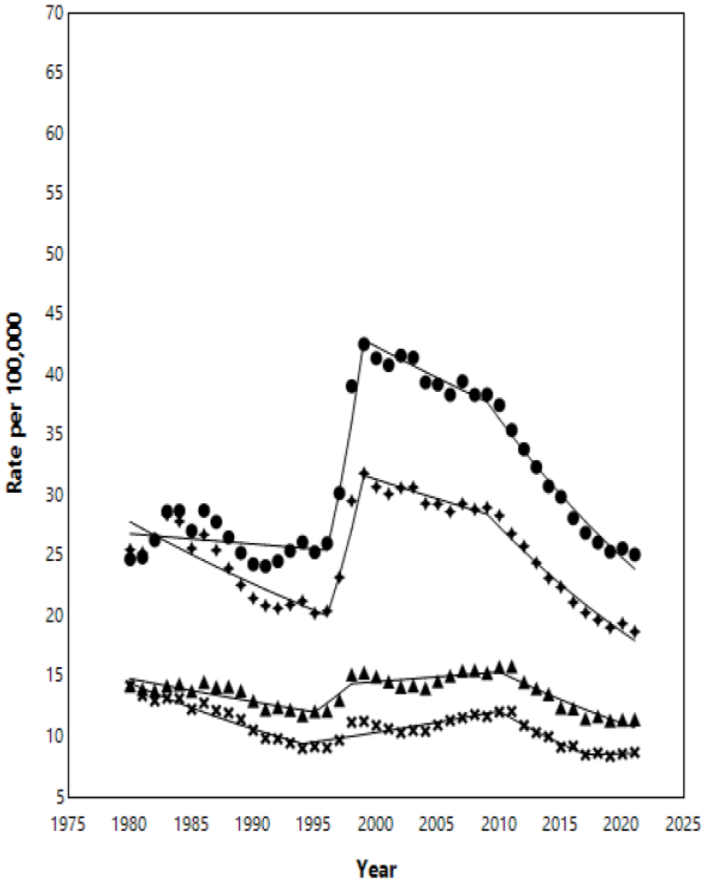

● Male / All ages – 3 Joinpoints
1980–1996 APC = −0.3
1996–1999 APC = 19.0*
1999–2009 APC = −1.3
2009–2021 APC = −3.7*

▲ Female / All ages – 3 Joinpoints
1980–1995 APC = −1.4*
1995–1998 APC = 6.2
1998–2010 APC = 0.5
2010–2021 APC = −3.2*

✚ Male / Age-standardized – 3 Joinpoints
1980–1996 APC = −2.0*
1996–1999 APC = 16.5*
1999–2009 APC = −1.1
2009–2021 APC = −3.8*

✕ Female / Age-standardized – 3 Joinpoints
1980–1994 APC = −3.0*
1994–2010 APC = 1.6
2010–2017 APC = −4.9
2017–2021 APC = 0.2

34. Saga Prefecture

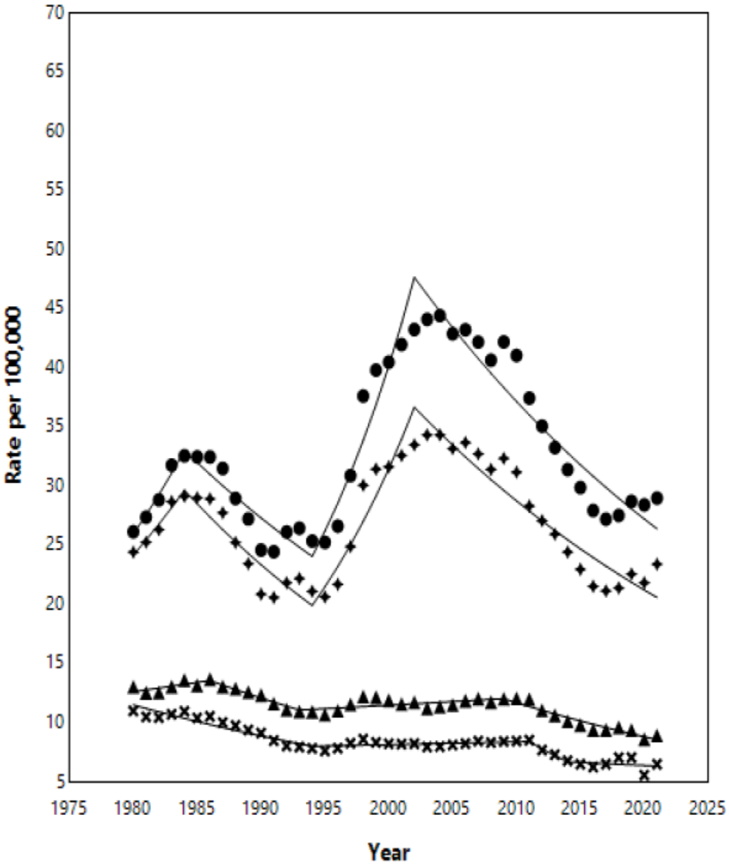

● Male / All ages – 3 Joinpoints
1980–1984 APC = 6.2*
1984–1994 APC = −3.1*
1994–2002 APC = 8.9*
2002–2021 APC = −3.1*

▲ Female / All ages – 3 Joinpoints
1980–1986 APC = 1.2
1986–1993 APC = −2.9*
1993–2009 APC = 0.5*
2009–2021 APC = −2.8*

✚ Male / Age-standardized – 3 Joinpoints
1980–1984 APC = 5.3*
1984–1994 APC = −3.9*
1994–2002 APC = 7.9*
2002–2021 APC = −3.0*

✕ Female / Age-standardized – 3 Joinpoints
1980–1994 APC = −2.6*
1994–2011 APC = 0.3
2011–2014 APC = −7.4
2014–2021 APC = −0.8

35. Saitama Prefecture

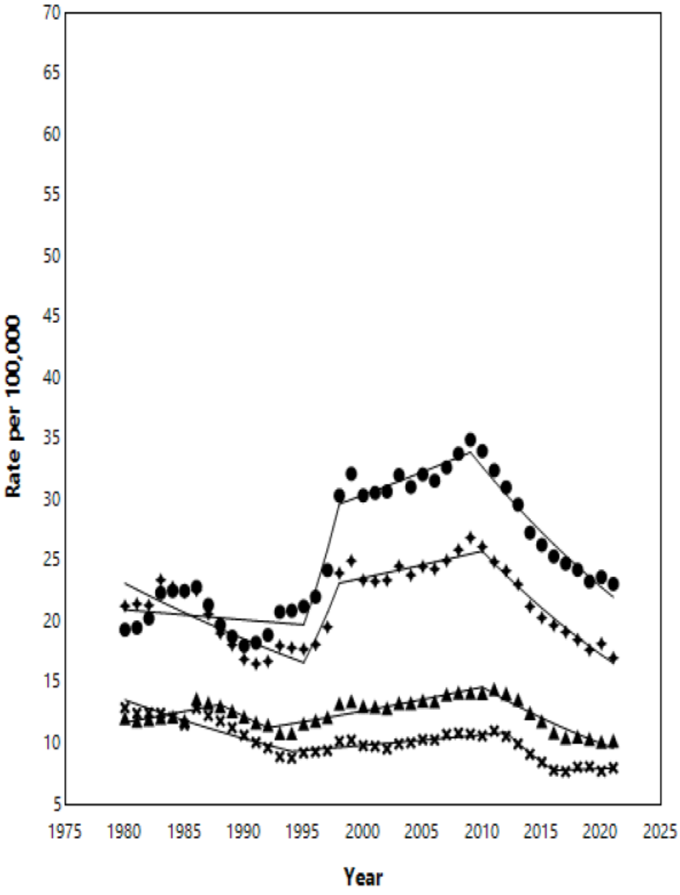

● Male / All ages – 3 Joinpoints
1980–1995 APC = −0.4
1995–1998 APC = 14.6*
1998–2009 APC = 1.2
2009–2021 APC = −3.5*

▲ Female / All ages – 3 Joinpoints
1980–1988 APC = 1.5*
1988–1992 APC = −3.8*
1992–2010 APC = 1.5*
2010–2021 APC = −3.8*

✚ Male / Age-standardized – 3 Joinpoints
1980–1995 APC = −2.2*
1995–1998 APC = 11.7*
1998–2010 APC = 0.9
2010–2021 APC = −3.9*

✕ Female / Age-standardized – 3 Joinpoints
1980–1994 APC = −2.6*
1994–2012 APC = 0.9*
2012–2016 APC = −8.1*
2016–2021 APC = 0.5

36. Shiga Prefecture

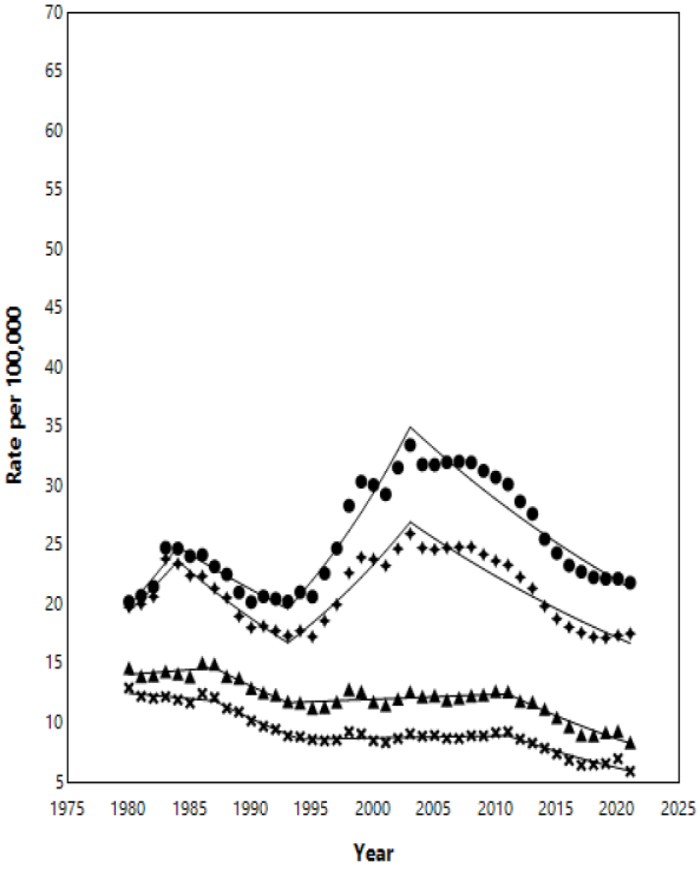

● Male / All ages – 3 Joinpoints
1980–1984 APC = 5.7*
1984–1993 APC = −2.6*
1993–2003 APC = 5.9*
2003–2021 APC = −2.7*

▲ Female / All ages – 3 Joinpoints
1980–1987 APC = 0.6
1987–1993 APC = −3.7*
1993–2011 APC = 0.3*
2011–2021 APC = −4.0*

✚ Male / Age-standardized – 3 Joinpoints
1980–1984 APC = 5.1*
1984–1993 APC = −3.8*
1993–2003 APC = 4.9*
2003–2021 APC = −2.6*

✕ Female / Age-standardized – 3 Joinpoints
1980–1987 APC = −0.7
1987–1994 APC = −4.5*
1994–2011 APC = 0.2
2011–2021 APC = −4.0*

37. Shimane Prefecture

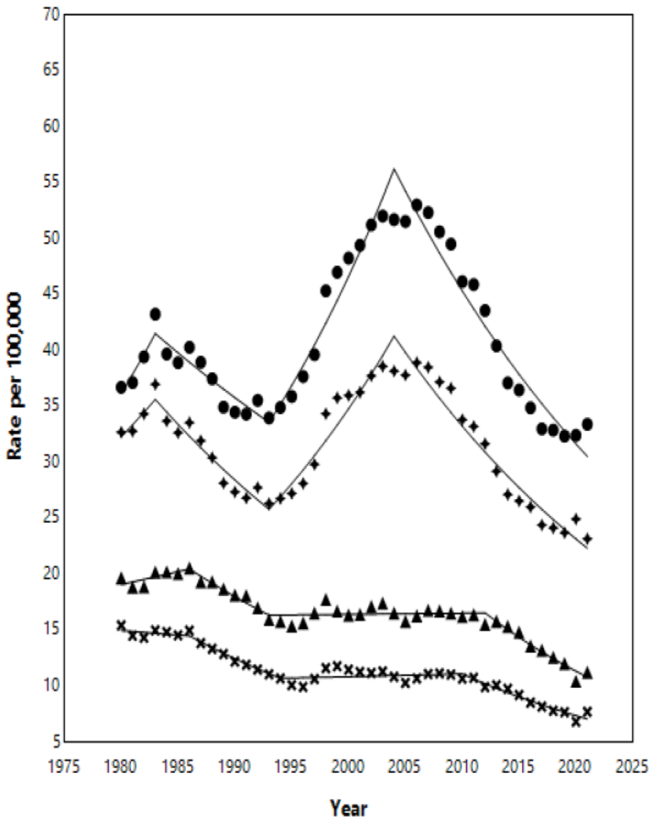

● **Male / All ages – 3 Joinpoints**
1980–1983 APC = 4.7
1983–1993 APC = −2.1
1993–2004 APC = 4.8
2004–2021 APC = −3.5*

▲ **Female / All ages – 3 Joinpoints**
1980–1986 APC = 1.2
1986–1993 APC = −3.2*
1993–2012 APC = 0.1
2012–2021 APC = −4.6*

✚ **Male / Age-standardized – 3 Joinpoints**
1980–1983 APC = 3.3
1983–1993 APC = −3.2
1993–2004 APC = 4.4
2004–2021 APC = −3.6*

✕ **Female / Age-standardized – 3 Joinpoints**
1980–1986 APC = −0.5
1986–1994 APC = −3.7
1994–2010 APC = 0.2
2010–2021 APC = −4.0*

38. Shizuoka Prefecture

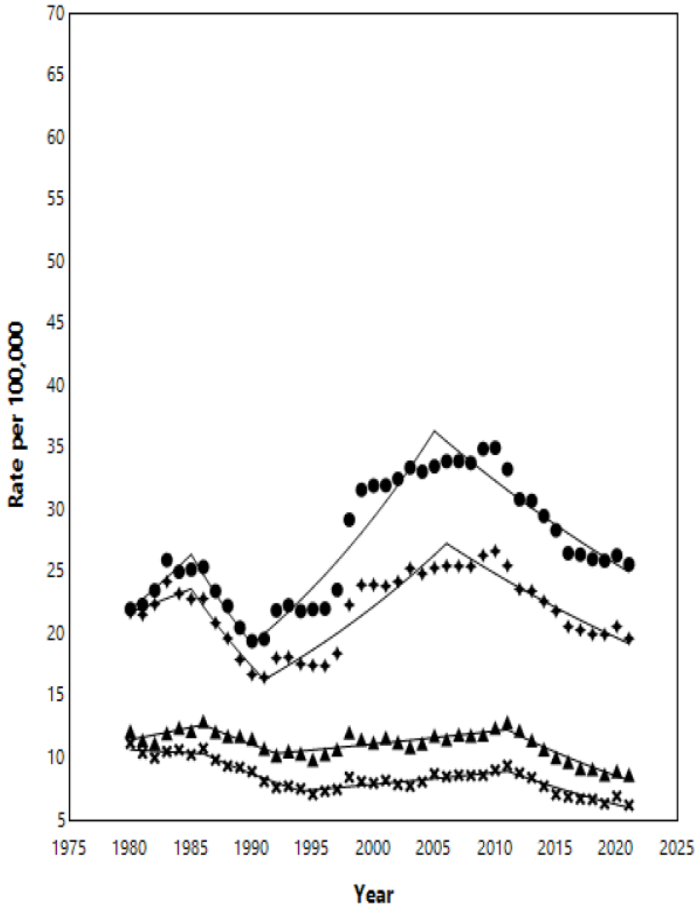

● **Male / All ages – 3 Joinpoints**
1980–1985 APC = 3.7*
1985–1990 APC = −6.2*
1990–2005 APC = 4.4*
2005–2021 APC = −2.3*

▲ **Female / All ages – 3 Joinpoints**
1980–1986 APC = 1.7*
1986–1992 APC = −3.3*
1992–2011 APC = 0.9*
2011–2021 APC = −4.0*

✚ **Male / Age-standardized – 3 Joinpoints**
1980–1985 APC = 1.7
1985–1991 APC = −6.0*
1991–2006 APC = 3.5*
2006–2021 APC = −2.3*

✕ **Female / Age-standardized – 3 Joinpoints**
1980–1986 APC = −0.5
1986–1994 APC = −4.2
1994–2011 APC = 1.2*
2011–2021 APC = −3.9*

39. Tochigi Prefecture

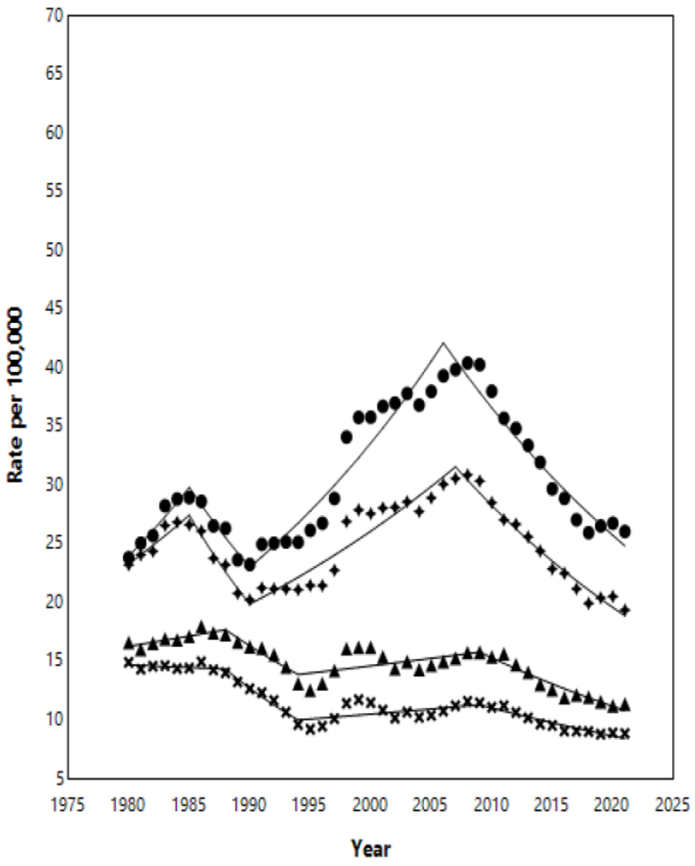

● Male / All ages – 3 Joinpoints
1980–1985 APC = 4.5*
1985–1990 APC = −5.0*
1990–2006 APC = 3.9*
2006–2021 APC = −3.5*

▲ Female / All ages – 3 Joinpoints
1980–1988 APC = 1.0
1988–1994 APC = −4.0*
1994–2009 APC = 0.9*
2009–2021 APC = −3.1*

✚ Male / Age-standardized – 3 Joinpoints
1980–1985 APC = 3.3*
1985–1990 APC = −6.3*
1990–2007 APC = 2.8*
2007–2021 APC = −3.6*

✕ Female / Age-standardized – 3 Joinpoints
1980–1988 APC = −0.3
1988–1994 APC = −5.8*
1994–2009 APC = 0.8*
2009–2021 APC = −2.4*

40. Tokushima Prefecture

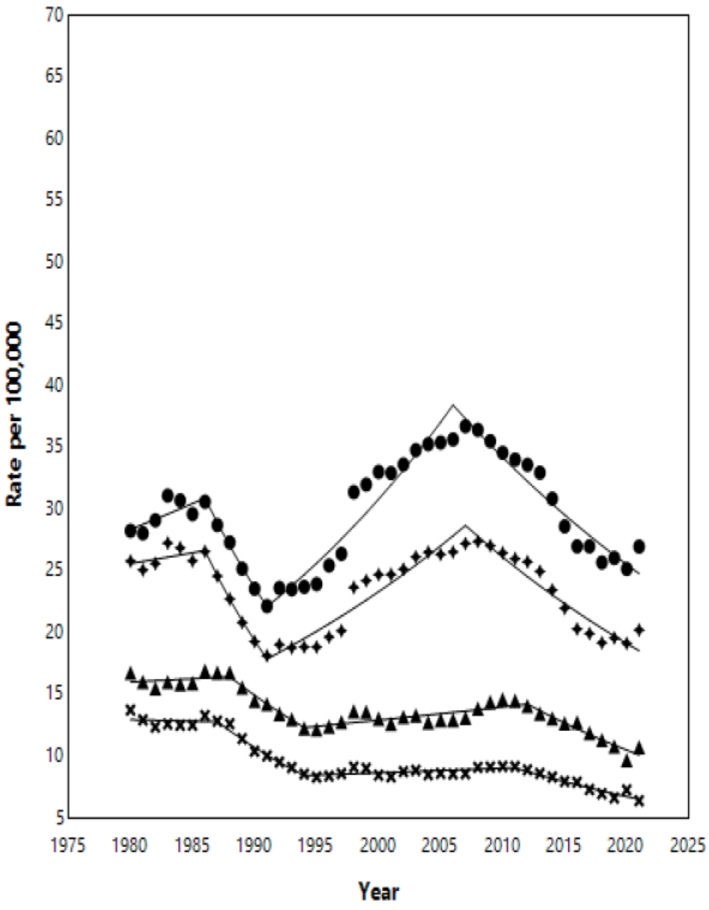

● Male / All ages – 3 Joinpoints
1980–1986 APC = 1.4
1986–1991 APC = −6.5*
1991–2006 APC = 3.8*
2006–2021 APC = −2.9*

▲ Female / All ages – 3 Joinpoints
1980–1988 APC = 0.3
1988–1994 APC = −4.7*
1994–2012 APC = 0.8*
2012–2021 APC = −3.7*

✚ Male / Age-standardized – 3 Joinpoints
1980–1986 APC = 0.7
1986–1991 APC = −7.7*
1991–2007 APC = 3.0*
2007–2021 APC = −3.1*

✕ Female / Age-standardized – 3 Joinpoints
1980–1987 APC = −0.2
1987–1994 APC = −5.8*
1994–2011 APC = 0.4*
2011–2021 APC = −3.3*

41. Tokyo Prefecture

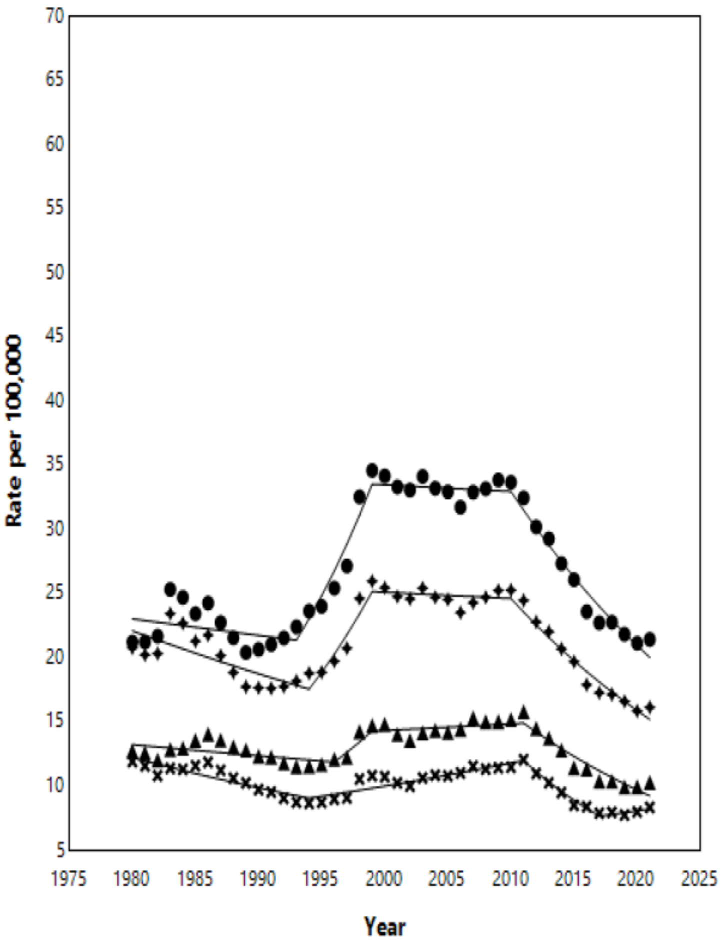

● Male / All ages – 3 Joinpoints
1980–1993 APC = −0.6
1993–1999 APC = 7.8*
1999–2010 APC = −0.2
2010–2021 APC = −4.4*

▲ Female / All ages – 3 Joinpoints
1980–1996 APC = −0.7
1996–1999 APC = 6.4
1999–2011 APC = 0.4
2011–2021 APC = −4.7*

✚ Male / Age-standardized – 3 Joinpoints
1980–1994 APC = −1.6*
1994–1999 APC = 7.5*
1999–2010 APC = −0.2
2010–2021 APC = −4.3*

✕ Female / Age-standardized – 3 Joinpoints
1980–1994 APC = −2.1*
1994–2011 APC = 1.7*
2011–2017 APC = −7.2*
2017–2021 APC = 1.7

42. Tottori Prefecture

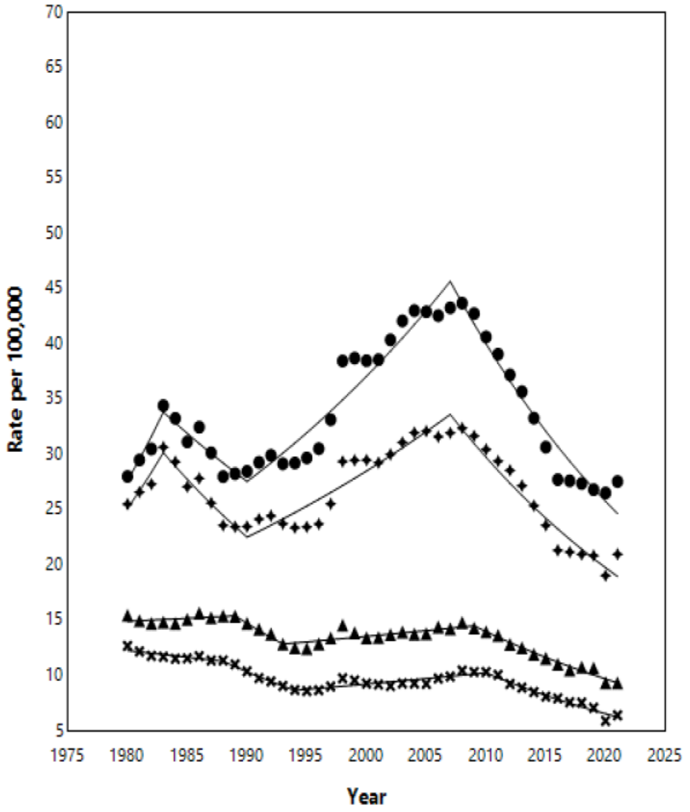

● Male / All ages – 3 Joinpoints
1980–1983 APC = 7.0*
1983–1990 APC = −2.9*
1990–2007 APC = 3.0*
2007–2021 APC = −4.3*

▲ Female / All ages – 3 Joinpoints
1980–1989 APC = 0.4
1989–1993 APC = −4.4*
1993–2009 APC = 0.8*
2009–2021 APC = −3.6*

✚ Male / Age-standardized – 3 Joinpoints
1980–1983 APC = 6.3*
1983–1990 APC = −4.1*
1990–2007 APC = 2.4*
2007–2021 APC = −4.0*

✕ Female / Age-standardized – 3 Joinpoints
1980–1988 APC = −1.0
1988–1994 APC = −4.3*
1994–2010 APC = 1.1*
2010–2021 APC = −4.4*

43. Toyama Prefecture

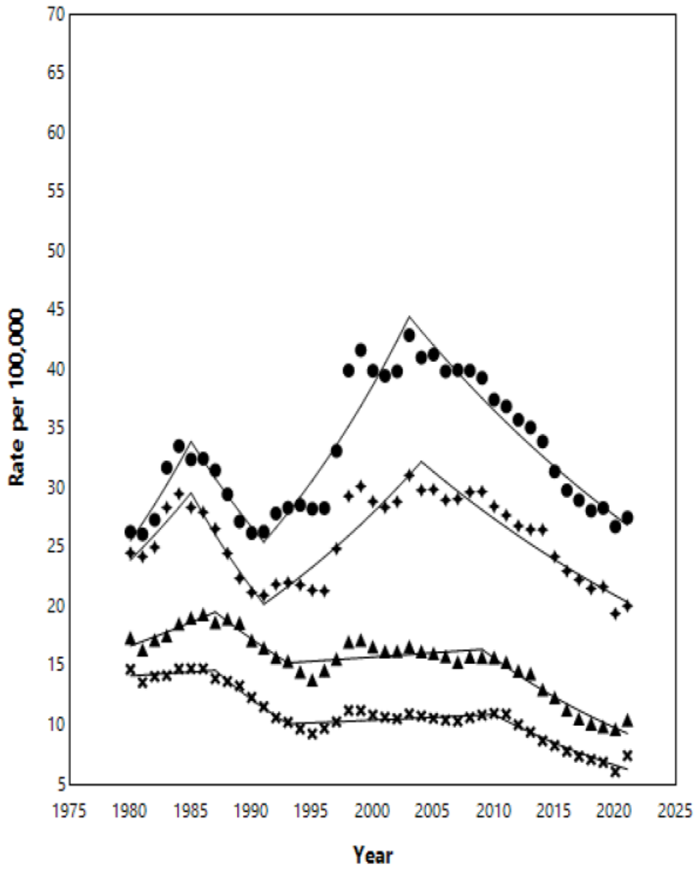

● **Male / All ages – 3 Joinpoints**
1980–1985 APC = 5.8*
1985–1991 APC = −4.7*
1991–2003 APC = 4.8*
2003–2021 APC = −2.8*

▲ **Female / All ages – 3 Joinpoints**
1980–1987 APC = 2.3*
1987–1993 APC = −4.1*
1993–2009 APC = 0.5
2009–2021 APC = −4.6*

✚ **Male / Age-standardized – 3 Joinpoints**
1980–1985 APC = 4.3*
1985–1991 APC = −6.2*
1991–2004 APC = 3.7*
2004–2021 APC = −2.7*

✕ **Female / Age-standardized – 3 Joinpoints**
1980–1987 APC = 0.5
1987–1993 APC = −6.0*
1993–2010 APC = 0.4
2010–2021 APC = −4.9*

44. Wakayama Prefecture

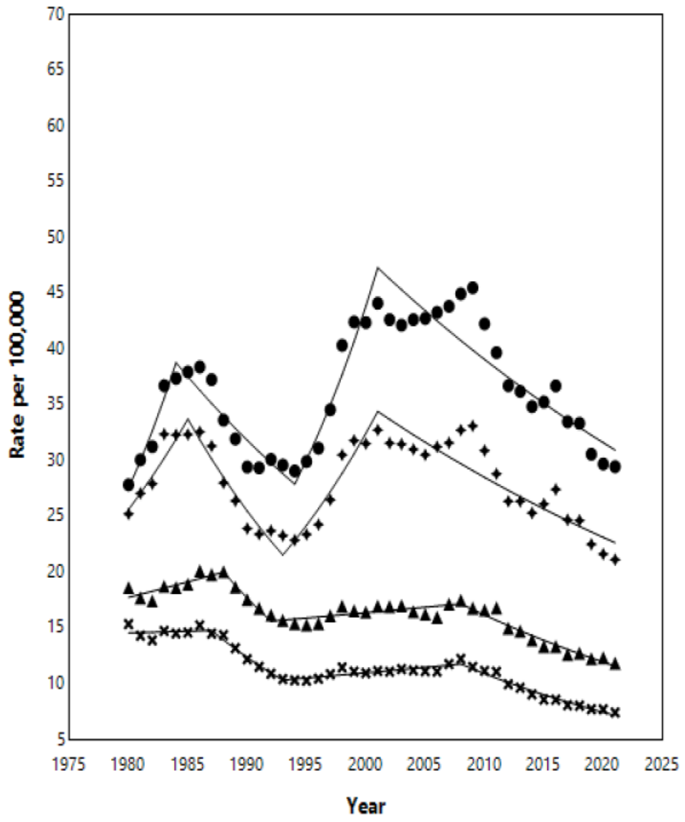

● **Male / All ages – 3 Joinpoints**
1980–1984 APC = 9.0*
1984–1994 APC = −3.2*
1994–2001 APC = 7.8*
2001–2021 APC = −2.1*

▲ **Female / All ages – 3 Joinpoints**
1980–1988 APC = 1.5*
1988–1992 APC = −6.0*
1992–2008 APC = 0.6*
2008–2021 APC = −3.0*

✚ **Male / Age-standardized – 3 Joinpoints**
1980–1985 APC = 5.7*
1985–1993 APC = −5.5*
1993–2001 APC = 6.0*
2001–2021 APC = −2.1*

✕ **Female / Age-standardized – 3 Joinpoints**
1980–1987 APC = 0.2
1987–1993 APC = −5.8*
1993–2008 APC = 0.9*
2008–2021 APC = −3.8*

45. Yamagata Prefecture

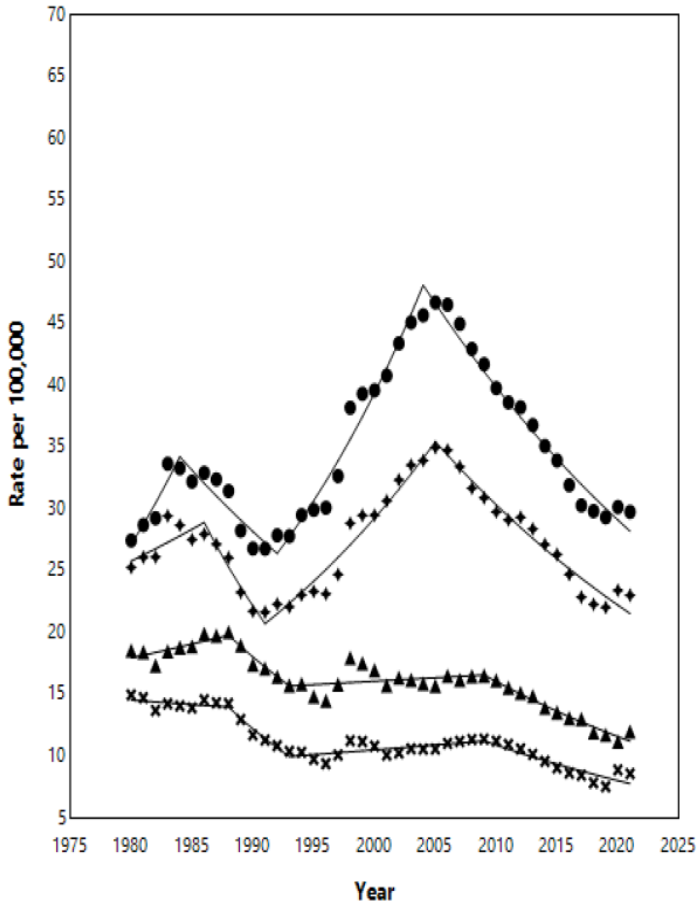

● Male / All ages – 3 Joinpoints
1980–1984 APC = 6.0*
1984–1992 APC = −3.2*
1992–2004 APC = 5.1*
2004–2021 APC = −3.1*

▲ Female / All ages – 3 Joinpoints
1980–1988 APC = 1.2*
1988–1993 APC = −4.6*
1993–2009 APC = 0.3
2009–2021 APC = −3.2*

✚ Male / Age-standardized – 3 Joinpoints
1980–1986 APC = 1.9*
1986–1991 APC = −6.5*
1991–2005 APC = 3.9*
2005–2021 APC = −3.1*

✕ Female / Age-standardized – 3 Joinpoints
1980–1988 APC = −0.5
1988–1993 APC = −6.5*
1993–2009 APC = 0.8*
2009–2021 APC = −3.0*

46. Yamaguchi Prefecture

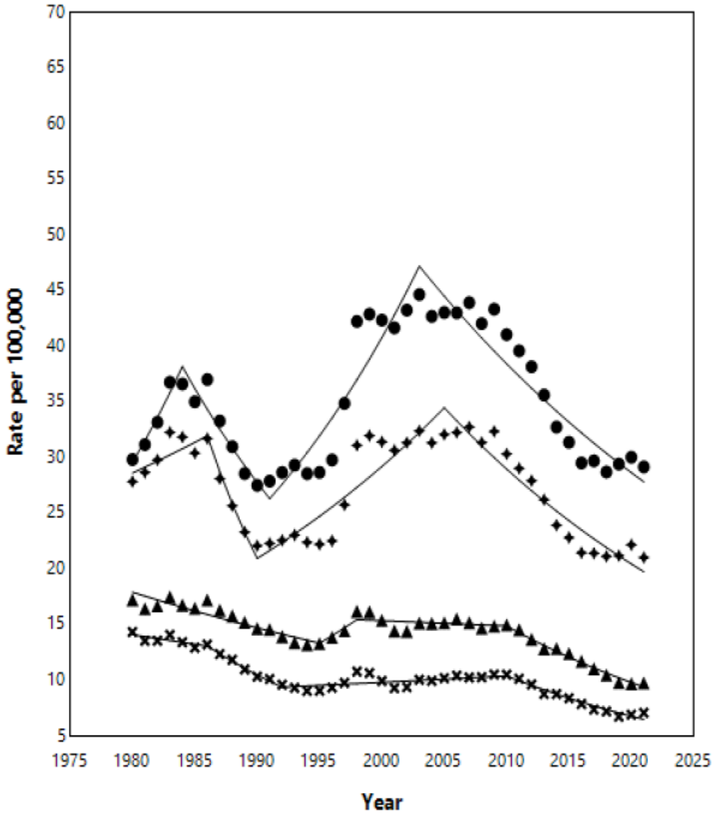

● Male / All ages – 3 Joinpoints
1980–1984 APC = 6.6*
1984–1991 APC = −5.2*
1991–2003 APC = 5.0*
2003–2021 APC = −2.9*

▲ Female / All ages – 3 Joinpoints
1980–1995 APC = −2.0*
1995–1998 APC = 5.0*
1998–2010 APC = −0.3
2010–2021 APC = −4.1*

✚ Male / Age-standardized – 3 Joinpoints
1980–1986 APC = 1.9
1986–1990 APC = −10.0*
1990–2005 APC = 3.4*
2005–2021 APC = −3.4*

✕ Female / Age-standardized – 3 Joinpoints
1980–1986 APC = −1.2
1986–1992 APC = −5.4*
1992–2010 APC = 0.6*
2010–2021 APC = −4.2*

47. Yamanashi Prefecture

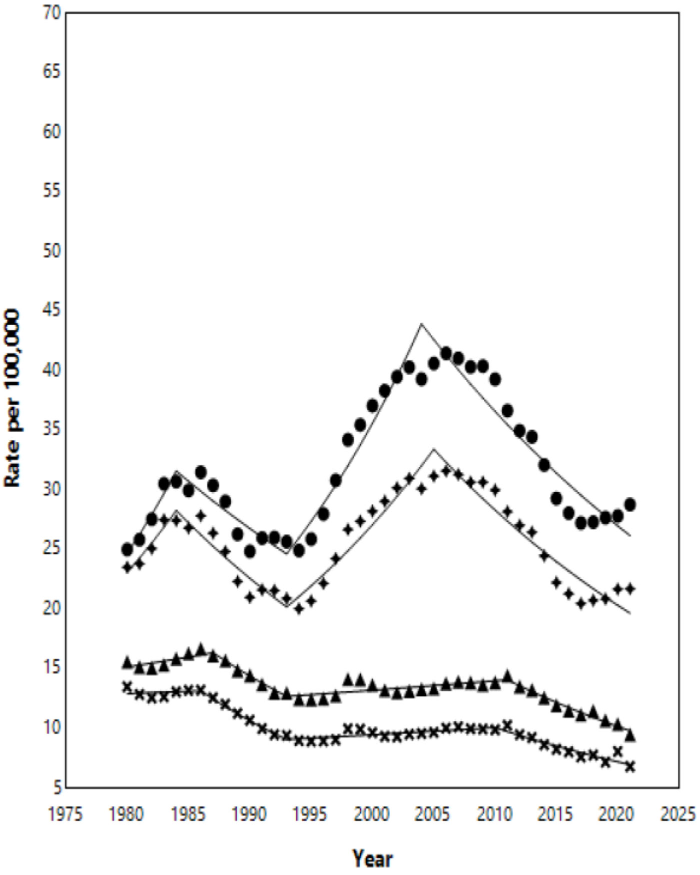

● Male / All ages – 3 Joinpoints
1980–1984 APC = 6.4*
1984–1993 APC = −2.7*
1993–2004 APC = 5.4*
2004–2021 APC = −3.0*

▲ Female / All ages – 3 Joinpoints
1980–1987 APC = 1.1*
1987–1993 APC = −4.2*
1993–2011 APC = 0.6*
2011–2021 APC = −3.6*

✚ Male / Age-standardized – 3 Joinpoints
1980–1984 APC = 5.2*
1984–1993 APC = −3.7*
1993–2005 APC = 4.3*
2005–2021 APC = −3.3*

✕ Female / Age-standardized – 3 Joinpoints
1980–1986 APC = 0.3
1986–1993 APC = −5.2*
1993–2010 APC = 0.6*
2010–2021 APC = −3.3*

Supplementary Figure 2. Age, Period and Cohort effects of 47 prefectures on the suicide death during 1982 -2021.

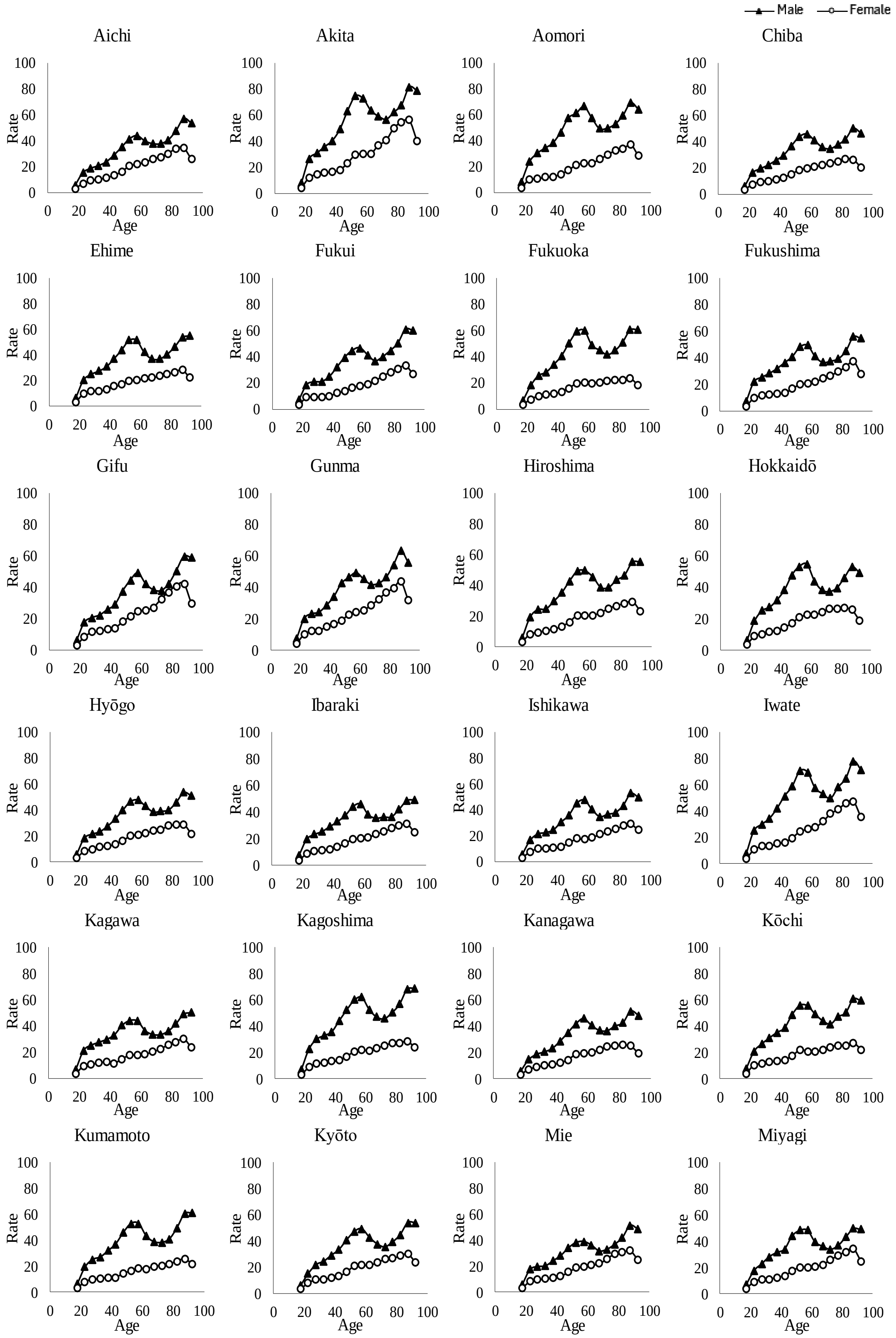

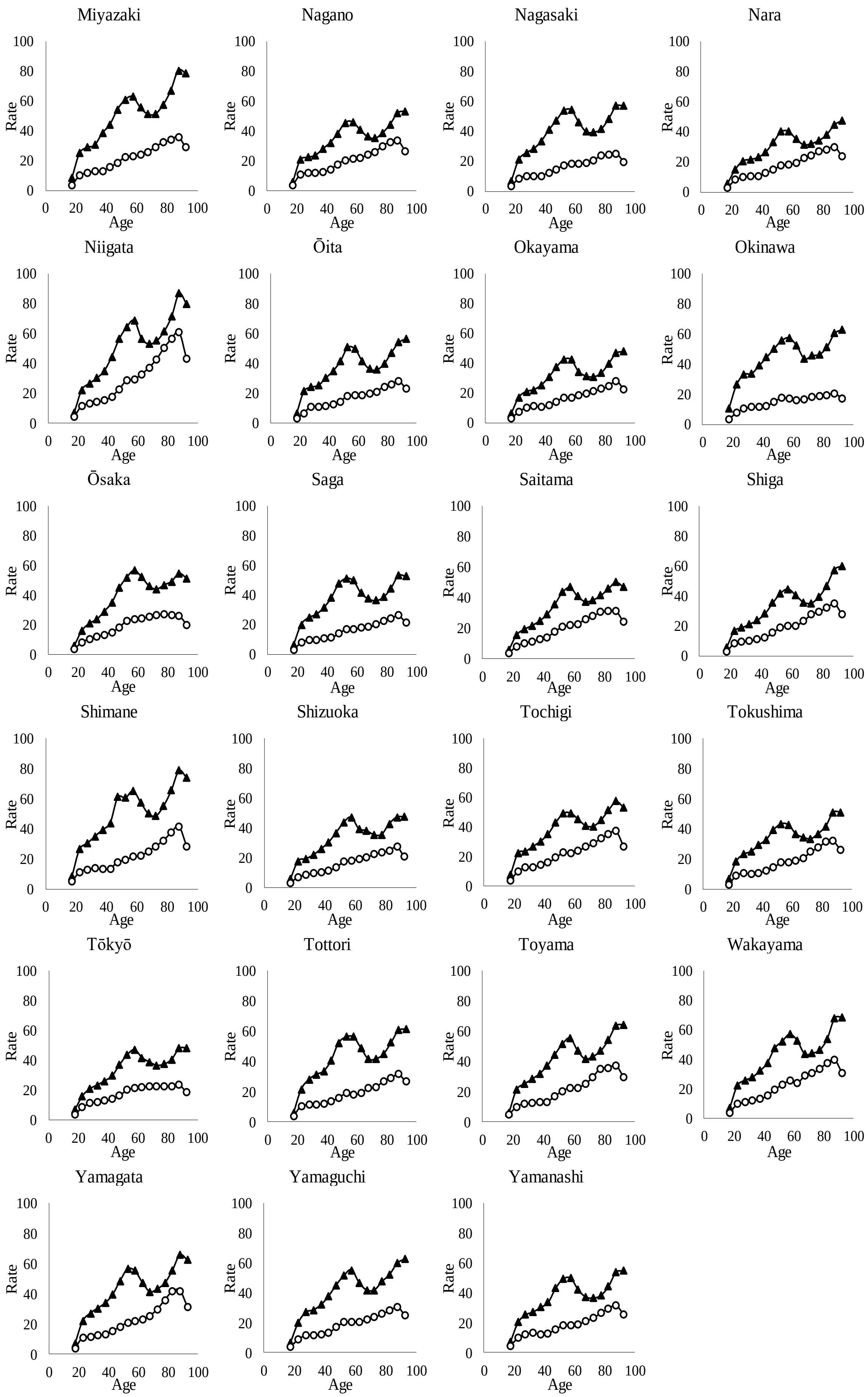
Miyazaki
Nagano
Nagasaki
Nara
Niigata
Ōita
Okayama
Okinawa
Ōsaka
Saga
Saitama
Shiga
Shimane
Shizuoka
Tochigi
Tokushima
Tōkyō
Tottori
Toyama
Wakayama
Yamagata
Yamaguchi
Yamanashi
Rate
Age

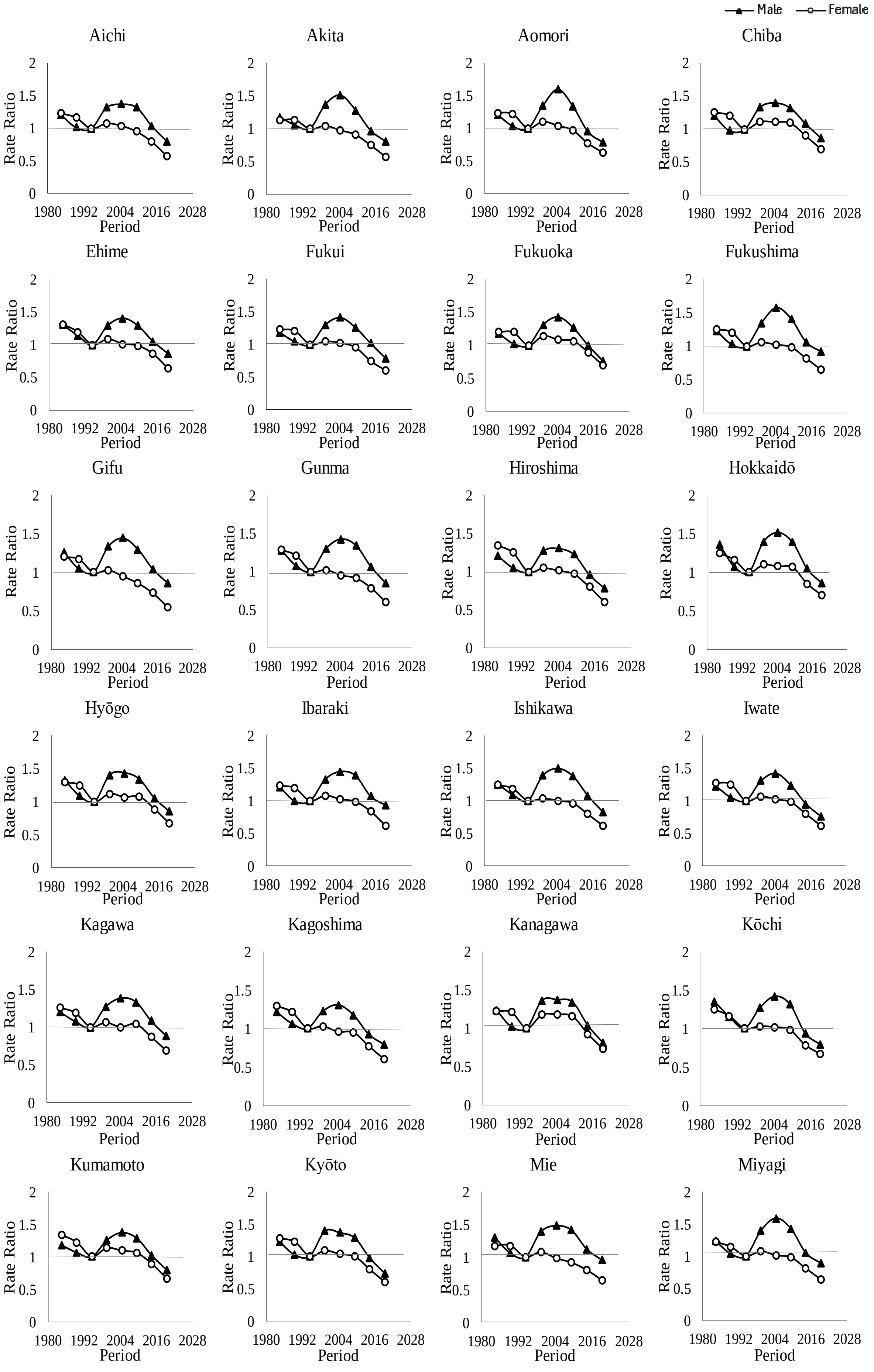

Male
Female
Aichi
Akita
Aomori
Chiba
Ehime
Fukui
Fukuoka
Fukushima
Gifu
Gunma
Hiroshima
Hokkaidō
Hyōgo
Ibaraki
Ishikawa
Iwate
Kagawa
Kagoshima
Kanagawa
Kōchi
Kumamoto
Kyōto
Mie
Miyagi
Rate Ratio
Period
0
0.5
1
1.5
2
1980
1992
2004
2016
2028

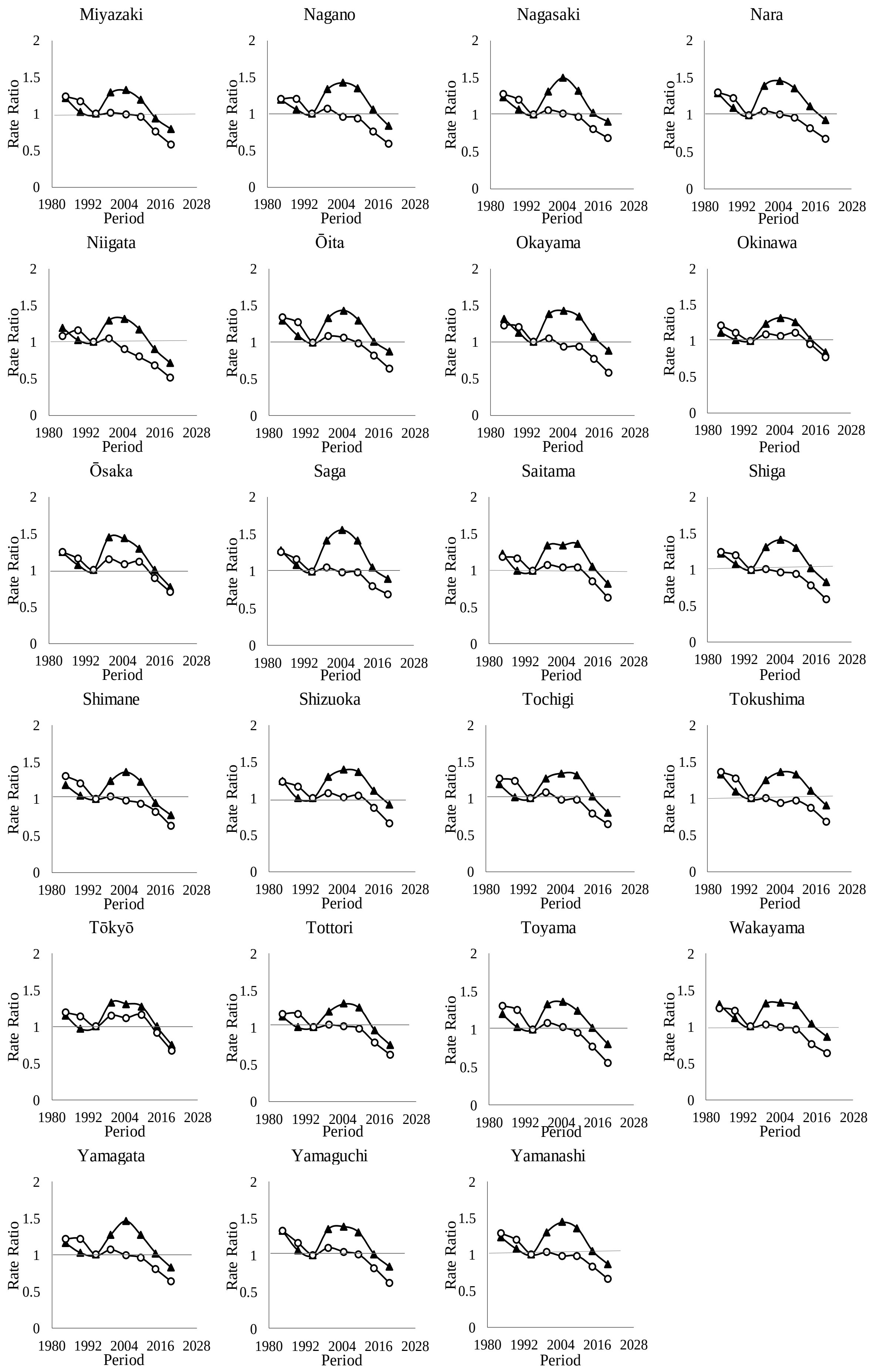
Miyazaki
Nagano
Nagasaki
Nara
Niigata
Ōita
Okayama
Okinawa
Ōsaka
Saga
Saitama
Shiga
Shimane
Shizuoka
Tochigi
Tokushima
Tōkyō
Tottori
Toyama
Wakayama
Yamagata
Yamaguchi
Yamanashi
Rate Ratio
0
0.5
1
1.5
2
Period
1980
1992
2004
2016
2028

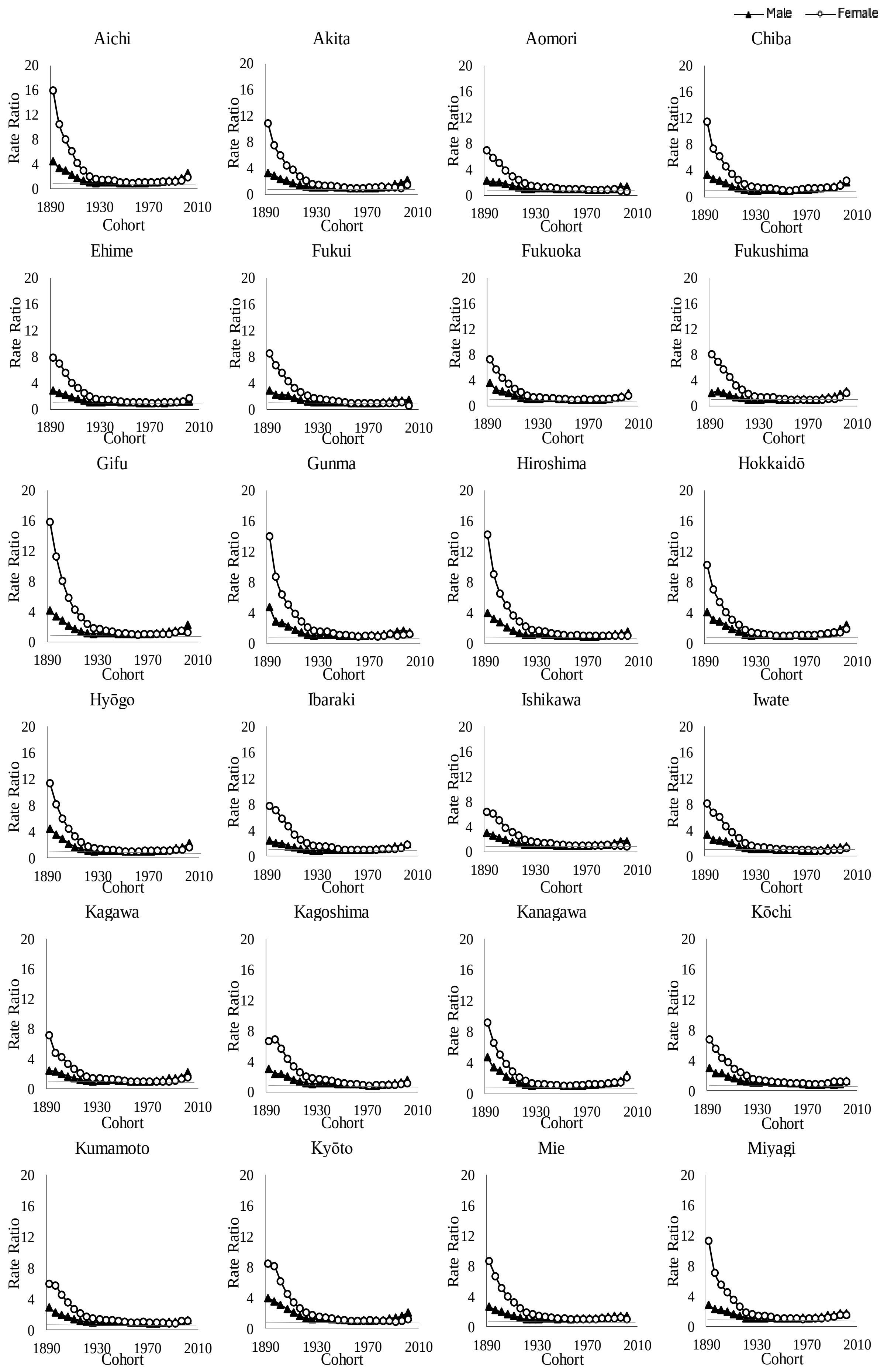

Male
Female
Aichi
Akita
Aomori
Chiba
Ehime
Fukui
Fukuoka
Fukushima
Gifu
Gunma
Hiroshima
Hokkaidō
Hyōgo
Ibaraki
Ishikawa
Iwate
Kagawa
Kagoshima
Kanagawa
Kōchi
Kumamoto
Kyōto
Mie
Miyagi
Rate Ratio
0
4
8
12
16
20
Cohort
1890
1930
1970
2010

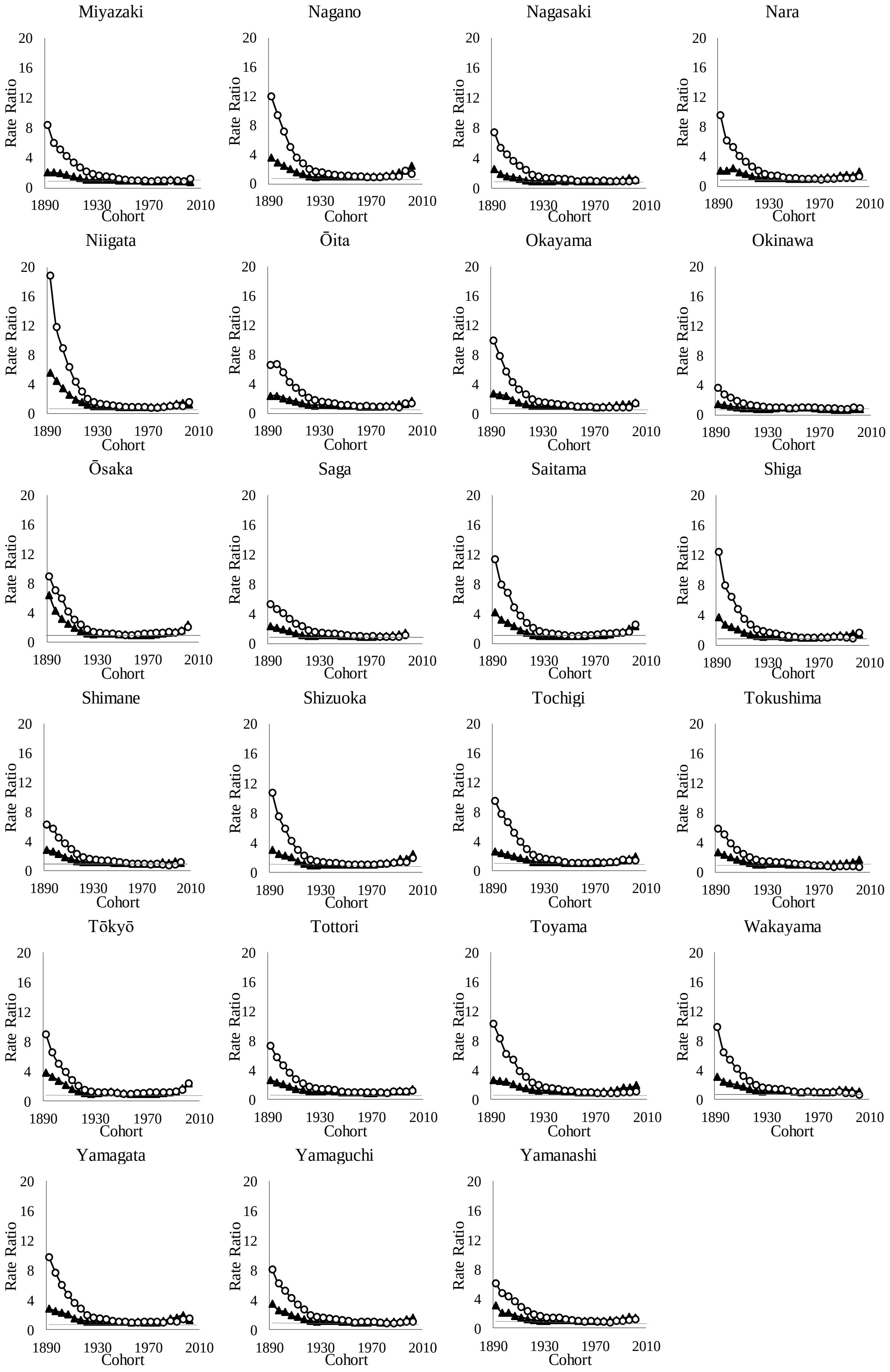
Miyazaki
Nagano
Nagasaki
Nara
Niigata
Ōita
Okayama
Okinawa
Ōsaka
Saga
Saitama
Shiga
Shimane
Shizuoka
Tochigi
Tokushima
Tōkyō
Tottori
Toyama
Wakayama
Yamagata
Yamaguchi
Yamanashi
Rate Ratio
0
4
8
12
16
20
Cohort
1890
1930
1970
2010
